# Ginseng and health outcomes: an umbrella review

**DOI:** 10.3389/fphar.2023.1069268

**Published:** 2023-07-03

**Authors:** Zhongyu Li, Yang Wang, Qing Xu, Jinxin Ma, Xuan Li, Yibing Tian, Yandong Wen, Ting Chen

**Affiliations:** ^1^ Department of Gastroenterology, Xiyuan Hospital, China Academy of Chinese Medical Sciences, Beijing, China; ^2^ Department of Chinese Medicine, Eye Hospital, China Academy of Chinese Medical Sciences, Beijing, China

**Keywords:** ginseng, health outcomes, meta-analysis, AMSTAR-2, umbrella review

## Abstract

**Background:** Ginseng consumption has been associated with various health outcomes. However, there are no review articles summarizing these reports.

**Methods:** PubMed, Embase, the Cochrane Library of Systematic Reviews, Scopus, CNKI and Wanfang databases were searched from inception to 31 July 2022. The Assessment of Multiple Systematic Reviews-2 (AMSTAR-2) and Grading of Recommendations Assessment, Development and Evaluation (GRADE) systems were used to assess the methodological quality and quality of evidence in each meta-analysis, and the results were summarized in a narrative form.

**Results:** Nineteen meta-analyses that met the eligibility criteria were identified from among 1,233 papers. The overall methodological quality was relatively poor, with only five studies being low-quality, and 14 critically low-quality. When compared with control treatments (mainly placebo), ginseng was beneficial for improving fatigue and physical function, sexual function, menopausal symptoms, metabolic indicators, inflammatory markers, unstable angina and respiratory diseases. Adverse events included gastrointestinal symptoms and potential bleeding; however, no serious adverse events were reported.

**Conclusion:** This umbrella review suggests that ginseng intake has beneficial therapeutic effects for diverse diseases. However, the methodological quality of studies needs to be improved considerably. In addition, it is imperative to establish the clinical efficacy of ginseng through high-quality randomized controlled trials.

## 1 Introduction

*Panax*, belonging to the *Acanthopanax* family, is a traditional botanical drug used worldwide (Wang et al., 2020; Li et al., 2022). Ginseng plants include several species in the *Panax* genus, such as *Panax ginseng C.A.Mey.* (Korean ginseng), Panax quinquefolius L. (American ginseng), and Panax notoginseng (Burkill) F.H.Chen (Chinese ginseng) (Wang et al., 2020). It also be divided into the following categories based on the processing method: fresh ginseng (under 4 years old, freshly consumed), white ginseng (between four and 6 years old, prepared by peeling and oven- or air-dried), sun ginseng (produced by steaming white ginseng under high temperatures and pressure), and red ginseng (6 years old, steamed without peeling) (Yun, 2001; Lü et al., 2009). Ginseng contains multiple chemically active ingredients (Kim, 2012) that exert positive pharmacological effects, including anti-diabetic (Yang et al., 2022), anti-inflammation and antioxidative stress (Bak et al., 2012; Yu et al., 2016), lowering-lipid levels (Liu et al., 2021), antitumor (Huang et al., 2022), and cardioprotective effects (Sun et al., 2016),etc. In addition, it has been noted that the daily consumption of ginseng could enhance human physical performance as well as quality of life (QoL) (Coleman et al., 2003; Bahrke et al., 2009). The therapeutic potential, diverse applications, and fast advancement of ginseng products has gained increased research attention in the correlation between ginseng and health outcomes. And numerous clinical trials have been conducted to validate the impact of ginseng on human health (Shergis et al., 2013; He et al., 2018; Chen et al., 2021). However, there is still a lack of a high-quality synthesis of the existing evidence in this field.

In recent years, umbrella review has been a novel approach evaluating the methodological quality and evidence of systematic reviews and meta-analyses (Aromataris et al., 2015). This approach has been implemented across various medical fields, involving psychotherapy (Leichsenring et al., 2022; Rabasco et al., 2022), nutritional supplementation (Fong et al., 2022), as well as herbal medicine (Zhang et al., 2022). Nevertheless, to our knowledge, there are no reviews assessing the methodological quality, and summarizing findings of ginseng. Therefore, this review aimed to provide a comprehensive overview of the correlation between ginseng and health outcomes. Based on this, we provide an evidence-based approach for ginseng use that would be useful for patients and may contribute to decision-making by clinicians.

## 2 Materials and methods

This umbrella review adhered to the guidelines of Preferred Reporting Items for Systematic Reviews and Meta-Analyses (PRISMA 2009) guidelines (Moher et al., 2009). There were no ethical requirements, as the analysis was based on published studies.

### 2.1 Literature search and eligibility criteria

The PubMed, Embase, the Cochrane Library of Systematic Reviews, Scopus, CNKI and Wanfang databases were searched from their inception until 31 July 2022. We used the following search terms: “ginseng”, “panax”, “systematic review”, or “meta-analysis” without language restrictions. Furthermore, the references of the involved reviews were screened to identify additional articles which fulfilled the inclusion criteria. The comprehensive search strategies are elaborated in Supplementary Table S1. Two reviewers (Y.W. and Q.X.) independently screened the titles and/or abstracts and reviewed full-text of articles for eligibility. Any disagreements were resolved by consulting a third researcher (YD. W.).

The selection of studies for inclusion was based on specific criteria related to population, interventions or exposures, comparators, outcomes, and study design: (1) population: adults aged ≥18 years; (2) interventions/exposures: oral ginseng administered either alone or as a supplement; (3) comparators: placebo, no treatment, or conventional therapy; (4) outcomes: any health outcomes (e.g., inflammatory markers, blood glucose levels); (5) study design: meta-analysis of randomized controlled trials (RCTs). To ensure the isolation of ginseng effects, studies that employed multi-herbal formulas were excluded. Furthermore, studies incorporating ginseng administration via topical application or injection were excluded due to different compositions and mechanisms. When multiple meta-analyses were available for the same topic and outcome, only the most recent meta-analysis was included in our analysis.

### 2.2 Data extraction

Two reviewers (J.X.M. and X.L.) independently extracted data using a tailored data extraction form. For each eligible study, we extracted the following details: the first author’s name, year of publication, country, disease status, study design, number of primary studies, sample size, intervention and comparison, types of ginseng, dosage, frequency, treatment duration, outcomes, and safety. In cases of disagreement, a third reviewer (T.C.) was consulted for resolution. To ensure data integrity, discrepancies arising from incomplete data were resolved through communication with the authors of the original research.

### 2.3 Assessment of methodological quality and quality of evidence

The Assessment of Multiple Systematic Reviews 2 (AMSTAR-2) checklist was used to assess the methodological quality of each meta-analysis (Shea et al., 2017). The AMSTAR-2 checklist comprises 16 items that are evaluated based on three rating options, namely, “yes” (Y), “partially yes” (PY), or “no” (N). The studies were evaluated based on their methodological quality and were categorized as “high”, “moderate”, “low” or “critically low”.

The Grading of Recommendations, Assessment, Development and Evaluation (GRADE) system was used to grade the quality of evidence (Guyatt et al., 2011). The risk of bias, inconsistency, indirectness, imprecision, and publication bias were assessed for each study, and the quality of evidence for each outcome was categorized as “high”, “moderate”, “low”, or “very low”.

Two researchers (J.X.Y and Y.B. T) independently evaluated the methodological and evidence quality. Any discrepancies were resolved through discussions and consultations with a third reviewer (Y.D. W.).

### 2.4 Statistical analysis

Health-related outcomes were extracted as descriptive summaries for every meta-analysis (Crichton et al., 2022; Zhang et al., 2022)). Estimated pooled effects—mean difference (MD), standardized mean difference (SMD), risk ratio (RR), and odds ratio (OR)—were extracted from the eligible meta-analyses, along with *p*-values and 95% confidence intervals (CIs) obtained using random-effects or fixed-effects models. I^2^ was used for evaluating heterogeneity. The evaluation of publication bias was conducted by funnel plots, as well as Egger’s and Begg’s tests. The compliance rates for items in the AMSTAR-2 checklist were measured for all meta-analyses and recorded as numbers and percentages of “Y”, “PY” or “N”. The data analysis and visualization were performed utilizing Excel 2016 (Microsoft Corporation, WA, United States).

## 3 Results

### 3.1 Literature search and characteristics of the included studies

**FIGURE 1:**
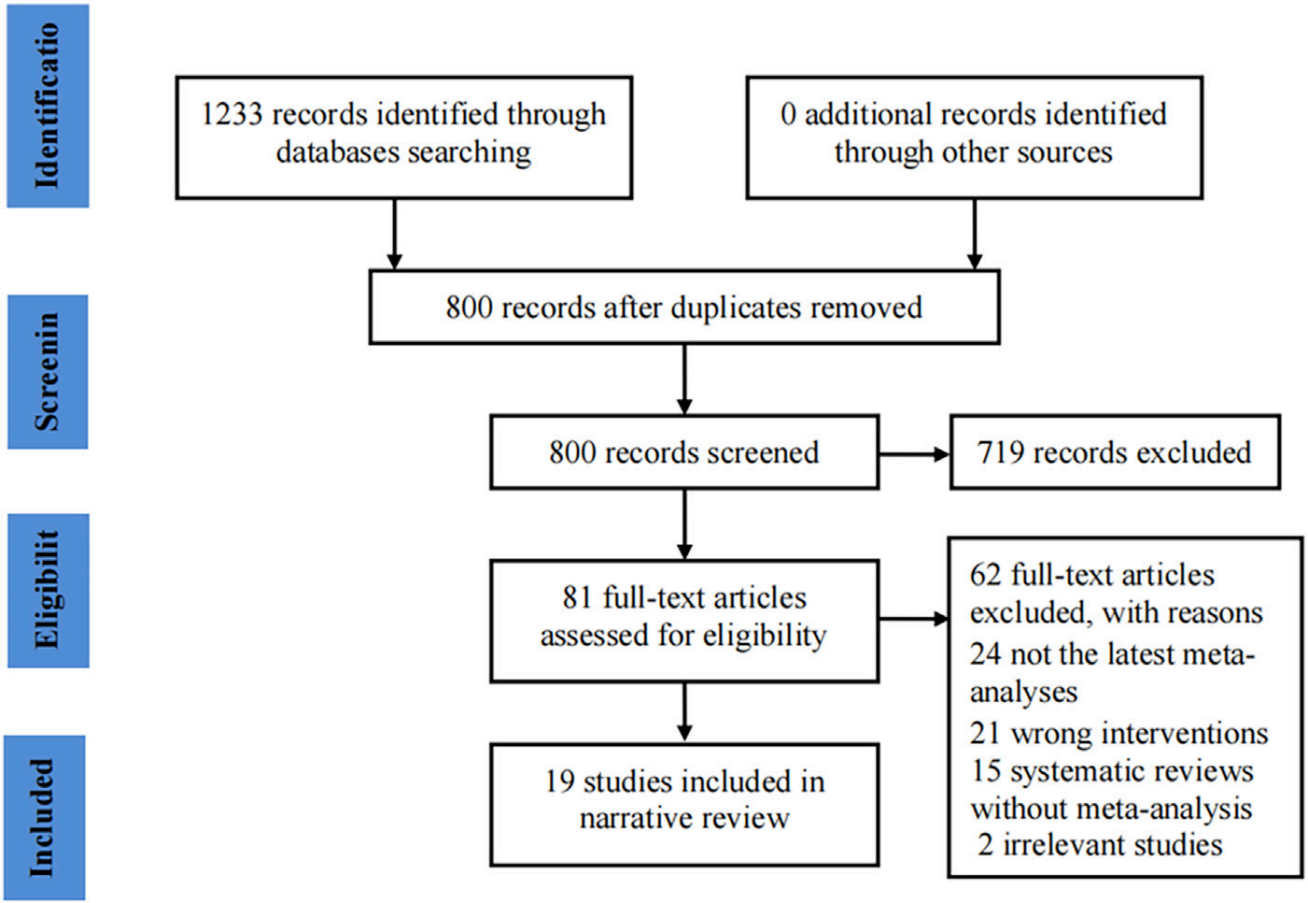
Flowchart of study selection for the umbrella review on ginseng consumption and health outcomes.

Our initial search identified 1,233 potentially eligible records. Of these, 800 records remained after removing duplicates. Subsequently, 719 records were excluded after reviewing the titles and abstracts. The full-texts of the remaining 81 records were evaluated, and 19 studies (Jang et al., 2008; Seida et al., 2011; Bach et al., 2016; Duan et al., 2018; Ghorbani and Mirghafourvand, 2019; Mohammadi et al., 2019; Saboori et al., 2019; Antonelli et al., 2020; Ghavami et al., 2020; Miraghajani et al., 2020; Lee et al., 2021; Sha’ari et al., 2021; Zhu et al., 2021; Ikeuchi et al., 2022; Lee et al., 2022; Luo and Huang, 2022; Naseri et al., 2022; Park et al., 2022; Zhu et al., 2022) were involved in the final analysis. The flow chart for the study selection process is shown in Figure 1.

Table 1 presents the key characteristics of the included meta-analyses. All meta-analyses were published from 2008 to 2022. These studies were published in peer-reviewed journals in eight geographical regions, including five studies each from Korea (Jang et al., 2008; Bach et al., 2016; Lee et al., 2021; Lee et al., 2022; Park et al., 2022) and Iran (Ghorbani and Mirghafourvand, 2019; Mohammadi et al., 2019; Saboori et al., 2019; Ghavami et al., 2020; Miraghajani et al., 2020); four from China (Duan et al., 2018; Zhu et al., 2021; Luo and Huang, 2022; Zhu et al., 2022); and one each from Italy (Antonelli et al., 2020), Canada (Seida et al., 2011), Japan (Ikeuchi et al., 2022), Australia (An et al., 2011), and Malaysia (Sha’ari et al., 2021). Eight studies had registered their protocols prior to publication (Antonelli et al., 2020; Lee et al., 2021; Sha’ari et al., 2021; Zhu et al., 2021; Ikeuchi et al., 2022; Lee et al., 2022; Luo and Huang, 2022; Park et al., 2022). The RCTs number involved in every meta-analysis varied between two and 28, and the total number of participants ranged between 123 and 2,503. Except for two studies conducted in healthy participants (Seida et al., 2011; Ikeuchi et al., 2022) and four that included both healthy controls and patients (Bach et al., 2016; Antonelli et al., 2020; Ghavami et al., 2020; Miraghajani et al., 2020), all remaining studies were conducted in patients with indications for ginseng use (Jang et al., 2008; Duan et al., 2018; Ghorbani and Mirghafourvand, 2019; Mohammadi et al., 2019; Saboori et al., 2019; Lee et al., 2021; Sha’ari et al., 2021; Zhu et al., 2021; Lee et al., 2022; Luo and Huang, 2022; Naseri et al., 2022; Park et al., 2022; Zhu et al., 2022). Moreover, 17 meta-analyses focused on placebo-controlled trials (Jang et al., 2008; Seida et al., 2011; Bach et al., 2016; Ghorbani and Mirghafourvand, 2019; Mohammadi et al., 2019; Saboori et al., 2019; Antonelli et al., 2020; Ghavami et al., 2020; Miraghajani et al., 2020; Lee et al., 2021; Sha’ari et al., 2021; Ikeuchi et al., 2022; Lee et al., 2022; Luo and Huang, 2022; Naseri et al., 2022; Park et al., 2022; Zhu et al., 2022), whereas two focused on eligible combinations (such as with usual medication) (Duan et al., 2018; Zhu et al., 2021). Most studies reported the type of ginseng, which mainly included *Panax ginseng*, *Panax notoginseng*, *Panax quinquefolius*, and red ginseng. The doses ranged from 5 mg to 8,000 mg. Two studies also reported the dosing frequency, which varied from one to three times a day (Lee et al., 2021; Luo and Huang, 2022). The treatment duration range was between 2 and 32 weeks. Regarding the risk of bias tools, the Cochrane risk of bias tool was employed in 15 studies (Duan et al., 2018; Ghorbani and Mirghafourvand, 2019; Mohammadi et al., 2019; Saboori et al., 2019; Antonelli et al., 2020; Ghavami et al., 2020; Miraghajani et al., 2020; Lee et al., 2021; Zhu et al., 2021; Ikeuchi et al., 2022; Lee et al., 2022; Luo and Huang, 2022; Naseri et al., 2022; Park et al., 2022; Zhu et al., 2022), three used the Jadad scale (Jang et al., 2008; Seida et al., 2011; Bach et al., 2016), and one used the McMaster Critical Appraisal Tool (Sha’ari et al., 2021).

**TABLE 1:** Characteristics of included studies.

Reference	Country	Health status	Intervention/comparation	Number of primary studies	Sample size	Duration	Registration information	Bias of risk assessment	Study design	Types of ginger	Doses	Frequency	Reported outcomes	Safety
Zhu et al. (2022)	China	Participants with underlying diseases	Ginseng/placebo	12	1,289	3–16 weeks	NR	Cochrane	RCTs	AG, PG, KG, RG	100–3000 mg/d	Twice	Disease-related fatigue	No
Naseri et al. (2022)	China	Type 2 diabetes mellitus	Ginseng/placebo	20	1,295	4–24 weeks	NR	Cochrane	RCTs of parallel or crossover design	Ginseng extract, PG, PQ	0.1–8 g/d	NR	BW, BMI, WC, FPG, OGTT, HbA1c, fasting insulin, HOMA-IR, TG, TC, LDL-C, HDL-C, SBP, DBP, HR, CRP, IL-6, TNF-α, ALT, AST, and GGT	No
Ikeuchi et al. (2022)	Japan	Adults	Panax Genus Plant or ginsenoside/placebo	5	123	Acute to 30days	UMIN-CTR (UMIN000043341)	Cochrane	RCTs of parallel or crossover design	PG, PN	5–1350 mg/d	NR	Exercise Endurance	No
Park et al. (2022)	Korea	Patients and healthy people	Ginseng/placebo	23	1,193	4–24 weeks	PROSPERO (CRD42020208191)	Cochrane	RCTs of parallel or crossover design	RG, FRG, PG, Ginseng berry	200mg to 8 g/d	NR	Glucose, insulin, HbA1c, SBP, DBP, BF, TC, TG, HDL-C, LDL-C	No
Luo and Huang (2022)	China	Cancer patients	Ginseng/placebo	7	1,335	4–16 weeks	PROSPERO (CRD42021241069)	Cochrane	RCTs	AG, KRG, CG	100–1000 mg/d	Once, twice, third	Cancer-Related Fatigue	Adverse Events
Lee et al. (2022)	South Korea	Menopausal women	Ginseng/placebo	15	1,192	2–16 weeks	Reviewregistry 1,342	Cochrane	RCTs of parallel or crossover design	KRG, AG, PG	200–3000 mg/d	NR	Menopausal symptoms, hot flashes, sexual function, QoL	Adverse events
Zhu et al. (2021)	China	Non-small cell lung cancer	Ginseng and its ingredients + chemotherapy/chemotherapy	28	2,503	21–120 days	PROSPERO (CRD: 42020220216)	Cochrane	RCTs	PG, Ginsenoside Rg3, Polysaccharide	NR	NR	ORR, DCR, QoL, leucopenia, thrombocytopenia, hemoglobin decline, myelosuppression, hepatotoxicity, alopecia, diarrhea, nausea and vomiting, 1-year survival rate, 2-year survival rate, immunity	No
Lee et al. (2021)	South Korea	Adult men with erectile dysfunction	Ginseng/placebo	9	587	4–12 weeks	Cochrane Library	Cochrane	RCTs of parallel or crossover design	KRG, TCMG	800–3000 mg/d	NR	Erectile function, ability to have intercourse reported by participants, sexual satisfaction	Adverse events
Sha’ari et al. (2021)	Selangor	Adult female	Panax ginseng/placebo	3	156	4–8 weeks	PROSPERO (CRD42021215136)	McMaster Critical Appraisal Tool	RCTs	PG	3 g/d	NR	Overall female sexual function, sexual arousal, sexual desire	No
Antonelli et al. (2020)	Italy	Adult subjects with SAURIs	Ginseng extract/placebo	8	2058	8–16 weeks	Open Science Framework (10.17605/OSF.IO/RW369)	Cochrane	RCTs	PQ, PG	NR	NR	Risk for developing an infection throughout the study period, duration of disease symptoms	No
Ghavami et al. (2020)	Iran	Adults	Ginseng/placebo	14	992	2–24 weeks	NR	Cochrane	RCTs of parallel or crossover design	AG, KRG, PG	0.75–6 g/d	NR	AST, ALT, GGT, ALP, ALB, BIL	No
Miraghajani et al. (2020)	Iran	Adults	Ginseng/placebo	11	457	4–12 weeks	NR	Cochrane	RCTs of parallel or crossover design	KRG, AG, Ginseng Root extract	3–8 g/d	NR	BMI, WC, BF%	No
Mohammadi et al. (2019)	Iran	Adults	Ginseng/placebo	8	409	3–32 weeks	NR	Cochrane	RCTs of parallel design	KRG, ginseng extract	300mg to 3 g/d	NR	IL-6, TNF-α, hs-CRP	No
Saboori et al. (2019)	Iran	Adults with any healthy status	Ginseng/placebo	7	420	4–28.8 weeks	NR	Cochrane	RCTs	RG, Ginseng extracts	300–4,500 mg/d	NR	CRP	No
Ghorbani and Mirghafourvand (2019)	Iran	Menopausal Women	Panax ginseng/placebo	5	531	6–16 weeks	NR	Cochrane	RCTs of parallel or crossover design	KRG, RG	200–6000 mg/d	NR	Sexual Function	Side events
Duan et al. (2018)	China	Unstable angina patients	PPCM and conventional medicine/conventional medicine	17	2,315	4–52 weeks	NR	Cochrane	RCTs	PN	NR	NR	End point, ECG, frequency and duration of angina attacks, dosage of nitroglycerin	Adverse events
Bach et al. (2016)	Korea	patients with underlying diseases or healthy people	Ginseng/placebo	12	659	4–12 weeks	NR	Jadad’s scale	RCTs	PG, PN, PQ	100–3000 mg/d	NR	Fatigue reduction, physical performance enhancement	No
Seida et al. (2011)	Canada	Healthy Adults	Ginseng/placebo	4	363	8–16 weeks	NR	Jadad’s scale	RCTs of parallel design	AG, PG	200 mg/d	twice	Incidence of common colds throughout the trial period, duration of colds	Adverse events
Jang et al. (2008)	South Korea	Patients with any type of erectile dysfunction	Red ginseng/placebo	7	363	4–12 weeks	NR	Jadad’s scale	RCTs of parallel or crossover design	RG	600mg to 1,000 mg/d	NR	Response rate, sexual functions	No

Abbreviations: PG: Panax ginseng; PN:Panax notoginseng; PQ: Panax quinquefolius; AG: American ginseng; KG: Korean ginseng; RG: Red ginseng; CG: Chinese ginseng; KRG: Korean Red ginseng; TCMG: tissue-cultured mountain ginseng; FRG: fermented red ginseng; HGE: Hydrolyzed ginseng extract; NR: Not reported; BF%: body fat percentage; BW: body weight; WC: waist circumference; BMI: Body Mass Index; FPG: fasting plasma glucose; OGTT: oral glucose tolerance test; HbA1c: hemoglobin A1c; HOMA-IR: homeostatic model assessment of insulin resistance; TG: triglyceride; TC:total cholesterol; LDL-C: low-density lipoprotein cholesterol; HDL-C: high-density lipoprotein cholesterol; SBP: systolic blood pressure; DBP: diastolic blood pressure; HR: heart rate; CRP: C-reactive protein; hs-CRP: high-sensitive C-Reactive Protein; IL-6: interlukin-6; TNF-α: tumor necrosis factor-α; ALT: alanine aminotransferase; AST: aspartate aminotransferase; GGT: gamma-glutamyl transferase; Qol: qulity of life; ORR: overall response rate; DCR: disease control rate; SAURIs: seasonal acute upper respiratory infections.

### 3.2 Quality of methodology and evidence

**FIGURE 2:**
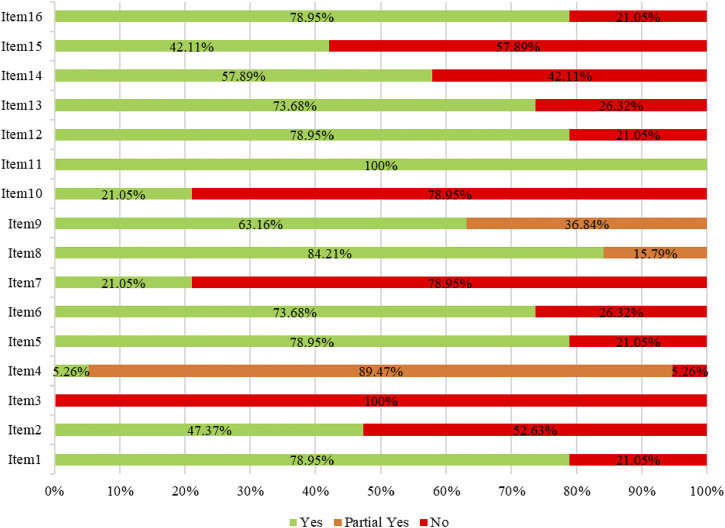
Methodological quality of included meta-analyses based on the AMSTAR-2 checklist.

The overall methodological quality of each study was evaluated based on the AMSTAR-2 checklist. The results showed that five studies were of low-quality and 14 were of critically low-quality (Table 2). The methodological quality limitations were mainly related to these items: item two, which requires the registration protocol to be established before conducting the review; item three, which necessitates an explanation of the chosen study designs for inclusion in the review; item four, which mandates the use of a comprehensive literature search strategy; item seven, which obliges the provision of a list of excluded studies and a justification for their exclusions; item 10, which establishes the reporting of funding sources for individual studies; and item 15, which entails the investigation of publication bias, (Figure 2).

**TABLE 2:** Results of methodological quality.

First author, year	Item1	Item2	Item3	Item4	Item5	Item6	Item7	Item8	Item9	Item10	Item11	Item12	Item13	Item14	Item15	Item16	Overall quality
Zhu et al. (2022)	Y	N	N	N	Y	Y	N	Y	Y	N	Y	N	N	N	Y	N	Critically low
Naseri et al. (2022)	Y	N	N	P	Y	Y	N	Y	Y	N	Y	Y	Y	Y	Y	Y	Critically low
Ikeuchi et al. (2022)	Y	Y	N	P	Y	Y	Y	Y	Y	N	Y	Y	Y	N	N	Y	Low
Park et al. (2022)	Y	Y	N	P	Y	Y	N	Y	Y	N	Y	Y	Y	N	Y	Y	Low
Luo and Huang (2022)	Y	Y	N	P	N	Y	N	P	P	N	Y	N	N	Y	N	Y	Critically low
Lee et al. (2022)	Y	Y	N	P	N	Y	N	Y	P	Y	Y	N	Y	N	N	Y	Critically low
Zhu et al. (2021)	Y	Y	N	P	Y	Y	N	Y	Y	N	Y	Y	Y	N	Y	Y	Low
Lee et al. (2021)	Y	Y	N	Y	Y	Y	Y	Y	Y	Y	Y	Y	Y	Y	N	Y	Low
Sha’ari et al. (2021)	Y	Y	N	P	Y	Y	N	Y	P	N	Y	Y	Y	N	N	Y	Critically low
Antonelli et al. (2020)	Y	Y	N	P	Y	Y	N	P	P	N	Y	Y	Y	N	N	Y	Critically low
Ghavami et al. (2020)	N	N	N	P	Y	Y	N	Y	Y	N	Y	Y	Y	Y	Y	Y	Critically low
Miraghajani et al. (2020)	N	N	N	P	Y	N	N	Y	Y	N	Y	Y	N	Y	Y	Y	Critically low
Mohammadi et al. (2019)	N	N	N	P	Y	N	Y	Y	Y	N	Y	Y	Y	Y	Y	N	Low
Saboori et al. (2019)	Y	N	N	P	Y	N	N	Y	Y	N	Y	Y	Y	N	Y	Y	Critically low
Ghorbani and Mirghafourvand (2019)	Y	N	N	P	N	N	N	Y	Y	N	Y	N	Y	Y	N	Y	Critically low
Duan et al. (2018)	Y	Y	N	P	Y	Y	N	P	Y	N	Y	Y	Y	Y	N	Y	Critically low
Bach et al. (2016)	Y	N	N	P	Y	N	N	Y	P	N	Y	Y	N	Y	N	Y	Critically low
Seida et al. (2011)	Y	N	N	P	Y	Y	N	Y	P	Y	Y	Y	Y	Y	N	N	Critically low
Jang et al. (2008)	N	N	N	P	N	Y	Y	Y	P	Y	Y	Y	N	Y	N	N	Critically low

Note Y: yes; N: no; P: partial yes. **Item 1:** Did the research questions and inclusion criteria for the review include the components of PICO?; **Item 2:** Did the report of the review contain an explicit statement that the review methods were established prior to the conduct of the review and did the report justify any significant deviations from the protocol?; **Item 3:** Did the review authors explain their selection of the study designs for inclusion in the review?; **Item 4:** Did the review authors use a comprehensive literature search strategy?; **Item 5:** Did the review authors perform study selection in duplicate?; **Item 6:** Did the review authors perform data extraction in duplicate?; **Item 7:** Did the review authors provide a list of excluded studies and justify the exclusions?; **Item 8:** Did the review authors describe the included studies in adequate detail?; **Item 9:** Did the review authors use a satisfactory technique for assessing the risk of bias (RoB) in individual studies that were included in the review?; **Item 10:** Did the review authors report on the sources of funding for the studies included in the review?; **Item 11:** If meta-analysis was performed did the review authors use appropriate methods for statistical combination of results?; **Item 12:** If meta-analysis was performed, did the review authors assess the potential impact of RoB in individual studies on the results of the meta-analysis or other evidence synthesis?; **Item 13:** Did the review authors account for RoB in individual studies when interpreting/discussing the results of the review?; **Item 14:** Did the review authors provide a satisfactory explanation for, and discussion of, any heterogeneity observed in the results of the review?; **Item 15:** If they performed quantitative synthesis did the review authors carry out an adequate investigation of publication bias (small study bias) and discuss its likely impact on the results of the review?; **Item 16:** Did the review authors report any potential sources of conflict of interest, including any funding they received for conducting the review?.

Of the 91 outcomes, the majority (81.32%) were associated with evidence of very low or low-quality, indicating that there was limited confidence that the estimated effects represented the true values. Evidence quality was considered moderate for the remaining 18.68% of outcomes. The GRADE levels were mostly downgraded owing to the risk of bias, inconsistency, and imprecision (Supplementary Table S2).

### 3.3 Therapeutic effects of ginseng

#### 3.3.1 Metabolic profiles

##### 3.3.1.1 Serum lipids

Three studies examined ginseng’s effects on serum lipids (Duan et al., 2018; Naseri et al., 2022; Park et al., 2022). In patients with metabolic syndrome (Mets), ginseng was found to reduce the total cholesterol (TC) (GRADE level: moderate), triglycerides (TG) (GRADE level: moderate), and low-density lipoprotein cholesterol (LDL-C) (GRADE level: moderate) when compared with placebo (Park et al., 2022). Although ginseng intake elevated high-density lipoprotein cholesterol (HDL-C) (GRADE level: moderate), the differences was not statistically significant (Park et al., 2022). In patients with prediabetes and type 2 diabetes mellitus (T2DM) patients, ginseng intake decreased TC (GRADE level: very low) but had no significant effect on TG (GRADE level: very low), LDL-C (GRADE level: very low), or HDL-C (GRADE level: very low) when compared to placebo (Naseri et al., 2022). When compared with lipid-lowering drugs alone, *Panax notoginseng* combined with lipid-lowering drugs significantly reduced TC, TG, and LDL-C levels and increased HDL-C levels, although the level of evidence was considered low (Duan et al., 2018).

##### 3.3.1.2 Blood glucose and insulin secretion

In terms of glucose metabolism, ginseng significantly reduced the serum concentrations of fasting plasma glucose (FPG) (GRADE level: very low) when compared with the placebo in patients with prediabetes and T2DM (Naseri et al., 2022). However, ginseng failed to reduce the oral glucose tolerance test results (OGTT) (GRADE level: very low) and hemoglobin A1c (HbA1c) levels (GRADE level: very low) (Naseri et al., 2022). In terms of insulin resistance and secretion, ginseng significantly reduced the Homeostatic Model Assessment of Insulin Resistance scores (GRADE level: low), but did not affect fasting insulin levels (GRADE level: very low) (Naseri et al., 2022).

##### 3.3.1.3 Anthropometric indices and body composition

In terms of weight management, ginseng supplementation had no significant effect on body weight (BW) (GRADE level: very low), body mass index (BMI) (GRADE level: low), or waist circumference (WC) (GRADE level: very low to low) when compared with the control treatment (mainly placebo) in adults/prediabetes and T2DM (Miraghajani et al., 2020; Naseri et al., 2022). However, there were contradictory results in body fat percentage (BF%). One study reported that ginseng had no effect on BF% in adults (GRADE level: very low) when compared to placebo (Miraghajani et al., 2020), whereas another study reported that ginseng significantly reduced BF% by 2.11% in patients with Mets (GRADE level: low) (Park et al., 2022).

##### 3.3.1.4 Blood pressure and heart rate

In terms of blood pressure, one study found that ginseng significantly reduced systolic blood pressure (SBP) (GRADE level: low), but failed to control diastolic blood pressure (DBP) (GRADE level: very low) in patients with metabolic diseases patients (Park et al., 2022). Another study showed that ginseng did not significantly affect SBP (GRADE level: very low) or DBP (GRADE level: very low) in patients with prediabetes and T2DM, but could increase the heart rate (GRADE level: low) (Naseri et al., 2022).

#### 3.3.2 Inflammatory markers and adipocytokines

Three studies evaluated the effects of ginseng on serum inflammatory parameters and adipocytokines (Mohammadi et al., 2019; Saboori et al., 2019; Naseri et al., 2022). One study indicated that ginseng reduced the serum interleukin 6 (IL-6) levels (GRADE level: very low) and tumor necrosis factor alpha (TNF-α) (GRADE level: very low) but had no effect on the high-sensitivity C-reactive protein (hs-CRP) levels (GRADE level: very low) in adults (Mohammadi et al., 2019). Similarly, in adults with any underlying health problem, ginseng did not reduce the serum levels of C-reactive protein (CRP) (GRADE level: very low) when compared to placebo (Saboori et al., 2019). Nevertheless, ginseng was associated with lower IL-6 levels (GRADE level: very low) and higher TNF-α levels (GRADE level: very low) than placebo, but had no effect on CRP (GRADE level: very low), lipocalin (GRADE level: very low) or leptin levels (GRADE level: very low) in patients with prediabetes and T2DM patients (Naseri et al., 2022).

#### 3.3.3 Fatigue and physical functioning

Four studies evaluated the effects of ginseng on physical functioning (Bach et al., 2016; Ikeuchi et al., 2022; Luo and Huang, 2022; Zhu et al., 2022). When compared with the placebo, ginseng was beneficial for reducing fatigue (GRADE level: moderate) (Bach et al., 2016), disease-related fatigue (GRADE level: moderate) (Zhu et al., 2022), and cancer-related fatigue (GRADE level: low) (Luo and Huang, 2022) in healthy people, patients with underlying diseases, or patients with cancer, respectively. Moreover, ginseng consumption was found to improve exercise endurance (GRADE level: low) in adults (Ikeuchi et al., 2022). However, there was no association between ginseng intake and physical performance (GRADE level: very low) (Bach et al., 2016).

#### 3.3.4 Sexual function and menopausal symptoms

Five studies evaluated the effects of ginseng on sexual function (Jang et al., 2008; Ghorbani and Mirghafourvand, 2019; Lee et al., 2021; Sha’ari et al., 2021; Lee et al., 2022). According to the Erectile Function Domain of the International Index of Erectile Function-15, ginseng had a trivial effect on erectile function (GRADE level: very low) (Lee et al., 2021). In addition, ginseng improved men’s self-reported ability to perform sexual intercourse (GRADE level: low) but had little effect on sexual satisfaction (GRADE level: low) (Lee et al., 2021). Another study showed that red ginseng was more effective in improving the erectile function than the placebo (GRADE level: low) (Jang et al., 2008). Among female patients with sexual dysfunction, ginseng failed to improve overall sexual function (GRADE level: very low), but had a positive effect in treating sexual arousal (GRADE level: very low) and sexual desire (GRADE level: very low) (Sha’ari et al., 2021). Similarly, ginseng failed to improve sexual function in menopausal women (GRADE level: very low) when compared with the placebo (Ghorbani and Mirghafourvand, 2019; Lee et al., 2022). However, ginseng could significantly reduce menopausal symptoms (GRADE level: low), hot flashes (GRADE level: low), and improve the QoL (GRADE level: moderate) in menopausal women (Lee et al., 2022).

#### 3.3.5 Respiratory diseases

Three studies evaluated the effects of ginseng supplements on respiratory disease (Seida et al., 2011; Antonelli et al., 2020; Zhu et al., 2021). Compared with the placebo, ginseng reduced the incidence of seasonal acute upper respiratory infections (SAURIs) during the intervention period (GRADE level: very low), but had no effect on their duration (GRADE level: very low) (Antonelli et al., 2020). One study reported a trend of ginseng intake reducing the incidence of common cold infections (GRADE level: low), although there was no statistically significant difference (Seida et al., 2011). In patients with non-small cell lung cancer (NSCLC), ginseng combined with chemotherapy was associated with improvements in the overall response rate (GRADE level: moderate), disease control rate (GRADE level: low), and QoL (GRADE level: low) (Zhu et al., 2021). In addition, ginseng supplementation had beneficial effects on chemotherapy-related complications; in particular, ginseng reduced the incidence of leukopenia (GRADE level: moderate), thrombocytopenia (GRADE level: moderate), myelosuppression (GRADE level: moderate), low hemoglobin levels (GRADE level: low), hepatotoxicity (GRADE level: moderate), nausea/vomiting (GRADE level: moderate), and diarrhea (GRADE level: moderate) (Zhu et al., 2021). Ginseng supplementation also significantly improved immune parameters, including the levels of CD3^+^ (GRADE level: low), CD4^+^ (GRADE level: very low), CD8^+^ (GRADE level: very low), and CD4+/CD8+ (GRADE level: very low) (Zhu et al., 2021). Importantly, ginseng supplementation also had positive effects on the 1-year survival rate (GRADE level: moderate) and 2-year survival rates (GRADE level: moderate) of patients with NSCLC (Zhu et al., 2021).

#### 3.3.6 Other indicators and diseases

Two studies evaluated the effects of ginseng on liver function markers (Ghavami et al., 2020; Naseri et al., 2022). When compared with the control group, orally administered ginseng had no effect on alanine aminotransferase (ALT) (GRADE level: low), aspartate aminotransferase (AST) (GRADE level: very low), gamma-glutamyl transferase (GGT) (GRADE level: low), alkaline phosphatase (ALP) (GRADE level: low), or albumin (ALB) (GRADE level: very low) in adults, although it was associated with a slight increase in bilirubin (BIL) levels (GRADE level: moderate) (Ghavami et al., 2020). Similarly, when compared with the placebo, ginseng exerted no effect on AST (GRADE level: very low), ALT (GRADE level: very low), or GGT (GRADE level: very low) in patients with prediabetes and T2DM patients (Naseri et al., 2022). Compared with conventional therapy, *P.notoginseng* saponin combined with traditional therapy effectively improved the values of electrocardiogram parameters (GRADE level: low) and reduced the occurrence of primary endpoint events (GRADE level: very low), frequency and duration of angina attacks (GRADE level: low to moderate), and dosage of nitroglycerin (GRADE level: low) in patients with unstable angina (Duan et al., 2018).

#### 3.3.7 Safety

Ten studies reported the safety of ginseng (Jang et al., 2008; Seida et al., 2011; Duan et al., 2018; Ghorbani and Mirghafourvand, 2019; Antonelli et al., 2020; Lee et al., 2021; Sha’ari et al., 2021; Ikeuchi et al., 2022; Lee et al., 2022; Luo and Huang, 2022). The most common adverse events were gastrointestinal symptoms (Jang et al., 2008; Seida et al., 2011; Duan et al., 2018; Ghorbani and Mirghafourvand, 2019; Sha’ari et al., 2021), including abdominal discomfort (Jang et al., 2008), constipation (Jang et al., 2008), dyspepsia (Ghorbani and Mirghafourvand, 2019; Sha’ari et al., 2021), and fecal occult blood (Duan et al., 2018). Some hematological adverse events were also reported, including neutropenia (Luo and Huang, 2022), vaginal bleeding (Ghorbani and Mirghafourvand, 2019; Sha’ari et al., 2021), subcutaneous hemorrhage, and rash (Duan et al., 2018). Other adverse reactions included headache (Jang et al., 2008), insomnia (Jang et al., 2008; Lee et al., 2022), palpitations (Lee et al., 2022), and flushing (Lee et al., 2022). In male patients with erectile dysfunction, ginseng had few side effects relative to placebo (RR: 1.45, 95% CI: 0.69 to 3.03; I^2^ = 0%; GRADE level: low) (Lee et al., 2021). The remaining studies used a descriptive approach and concluded that ginseng consumption appeared to be safe and well-tolerated (Antonelli et al., 2020; Lee et al., 2021; Ikeuchi et al., 2022).

## 4 Discussion

To our knowledge, this is the first review to assess the methodological quality and evidence of readily available meta-analyses on ginseng. Our review included 19 meta-analyses that provided a range of evidence related to the therapeutic effects of ginseng. The current findings suggest that ginseng is associated with improvements in physical strength, respiratory disease, sexual dysfunction and female menopausal symptoms, glucolipid metabolism, inflammatory markers, blood pressure, body weight management, and unstable angina. In addition, ginseng has a good safety profile.

Traditionally, ginseng has been used as a folk remedy for preventing and treating metabolic diseases, including diabetes, obesity, dyslipidemia, and cardiovascular disease (Yin et al., 2008). Several studies have showed the regulatory mechanisms underlying these effects of ginseng. For example, ginsenosides have been shown to improve insulin resistance and glucolipid metabolism through triggering IRS-1/PI3K/AKT as well as AMPK signaling pathways (Wang et al., 2022). It may also exert anti-obesity effects by regulating thermogenesis, lipogenesis, and lipolysis (Chen et al., 2017; Yoon et al., 2021). Ginseng may also exert beneficial pharmacological effects on blood pressure by mediating the inhibition of vascular myogenic activity (Qin et al., 2008). Furthermore, ginseng has been shown to exert cardioprotective effects via its antioxidant activity, by increasing coronary perfusion flow (Chen, 1996) and by enhancing contractile function during ischemic and reperfusion events (Deng and Zhang, 1991; Xie et al., 2006; Park et al., 2021). Ginseng also can negatively regulate pro-inflammatory cytokines expression and accelerate inflammation regression (Zhou et al., 2017; Kang et al., 2018). Ginseng consumption may aid in alleviating fatigue and help improving physical performance and exercise endurance by increasing SIRT1 deacetylase activity (Yang et al., 2018), improving energy metabolism, and inhibiting oxidative stress in skeletal muscles (Tan et al., 2013; Tan et al., 2014).

Ginseng may also have a positive impact on sexual function by promoting the release of endothelial nitric oxide, which improves penile hemodynamics and attenuates impairments in endothelial L-arginine-NO activity(Castela and Costa, 2016). This can directly influence the cavernous tissues and trigger erection via corporal smooth muscle relaxation (Castela and Costa, 2016; Ying et al., 2018). In addition, ginseng may have beneficial effects on preventing respiratory diseases. Indeed, ginseng can significantly amplify the serum antibody response to relevant vaccines (Song et al., 2010), inhibite lung inflammation, and reduce the infiltration of inflammatory cells into the lung tissue (Lee et al., 2018). Several studies on ginseng used in NSCLC patients have shown that ginseng extract inhibits tumor growth by altering the proliferation and morphology of tumor cells (Duan et al., 2017; Li et al., 2018; Lev-Ari et al., 2021), which highlights the potential for its widespread usage in clinical practice.

The active ingredients of ginseng have not been well reported in previous reviews. The quality and composition of its active ingredients may vary depending on numerous factors, including the plant species, cultivation methods, age at harvest, and the part of the plant used for extraction (Leung and Wong, 2010). Furthermore, there are studies reporting that the oral bioavailability of ginsenosides is very low. It may subject to poor permeability, low membrane permeability and biotransformation (Murugesan et al., 2022). Therefore, it is not possible to make appropriate recommendations on the dosage and dosing frequency of ginseng use based on current evidence. Although ginseng was not related to any significant side effects, it should not be used as a substitute for medication. Moreover, ginseng may not be appropriate for all populations, and it should only be administered under the care of physicians and/or nutritionists. The adverse events reported in most studies mainly including “gastrointestinal symptoms” and potential bleeding. However, patients and clinicians should remain aware of potential interactions between ginseng and conventional medicines, and further studies are required to identify any dangerous interactions (Izzo and Ernst, 2009; Dong et al., 2017). Because there is limited information on the safety of ginseng from clinical trials, it is difficult to draw definitive conclusions regarding its safety profile. Therefore, we hope that toxicological trials on ginseng, especially related to its long-term and frequent use, will be conducted in future. These studies will also help to provide more comprehensive safety data that can be used to standardize herbal medicine regimens (Qu et al., 2018).

In this review, we used the AMSTAR-2 checklist to evaluate the methodological quality of the included meta-analyses, and the findings indicated the existence of several possible constraints. Our findings indicate a need for significant improvements related to item two, three, four, seven, 10 and 15. As we know, registration and publication of protocols can promote the transparency and reproducibility of the meta-analyses (Page et al., 2018). It also reduces redundant efforts among diverse research teams (Sideri et al., 2018; Rombey et al., 2020). It is recommended that authors register their protocols in publicly available and open databases, such as the PROSPERO platform and Cochrane Library, to prevent possible bias in the study (Chien et al., 2012). The AMSTAR-2 checklist necessitates that review authors provide a rationale for their selection of a specific study design for meta-analysis (Shea et al., 2017), as study designs serve various purposes. In addition, a comprehensive literature search strategy is the foundation for conducting meta-analysis and guarantee the reliability of the findings. This also helps authors include all relevant studies and obtain accurate conclusions without the risk of selection bias (Qiu and Wang, 2016). Furthermore, review authors are required to provide a complete list, along with the rationale for their exclusion. This practice can enhance the transparency of the selection procedure and facilitate evaluating the integrity of the results. Authors should clearly disclose their sources of funding. Most studies indicate the absence of independent financial support, which is typically associated with financial conflicts of interest; for example, authors may present favorable outcomes and/or amplifying the effects of pharmaceuticals or devices supplied by industry sponsors (Lundh et al., 2018). Finally, publication bias may influence the pooled estimates by exaggerating the efficacy of a drug or downplaying the safety outcomes (Herrmann et al., 2017; Furuya-Kanamori et al., 2020). In this regard, funnel plots, Egger’s test, Begg’s test, and Macaskill’s test are all effective methods for assessing publication bias (Hayashino et al., 2005).

The GRADE system indicated that the evidence quality was very low to low. This emphasized the need for considerable improvements in future studies, which would help ensure that any clinical recommendations are based on high-quality data. The review revealed that the most frequent downgrade factor was the risk of bias. The primary cause was that RCTs lacked transparent or entire information on randomization, blinding, and allocation concealment. Hence, it is recommended that researchers directing upcoming investigations allocate more significant consideration toward the design framework and execution processes. Additionally, researchers should comply with basic guidelines for reporting clinical trials, such as—CONSORT statement (Schulz et al., 2010)—so as to provide better evidence to support their healthcare recommendations. Another downgrade factor was the significant heterogeneity across studies, which may be related to differences in the type of ginseng used and the dosage, dosing frequency, and treatment duration. Future RCTs should standardize the bioactive components of ginseng and provide detailed data related to the above parameters, as well as treatment adherence. Additional evidence is also required to verify the value of ginseng for clinical use in patients with high blood pressure and liver diseases. Other priority research areas include the anti-aging, immunomodulatory, and neuroprotective effects of ginseng; although there is plenty of evidence from animal and mechanistic studies supporting the effects of ginseng, its impact on humans has not been thoroughly investigated in clinical trials.

This review had several strengths and limitations. One strength of this review is that it synthesized evidence-based data from clinical practice, and the findings improved our understanding of the effects of ginseng use in the clinical setting. Notably, we adopted a rigorous study design that included assessments of methodological quality and quality of evidence employing AMSTAR-2 (Shea et al., 2017) and GRADE system (Guyatt et al., 2011) latest versions. The findings can guide future research and aid in making clinical decisions. However, although two trained researchers independently evaluated the quality of methodology and evidence in this review, any subjective biases could not be eliminated. Moreover, as most studies did not mention the side effects, which made it difficult to precisely evaluate ginseng safety in clinical practice.

## 5 Conclusion

Our umbrella review suggests that ginseng has beneficial effects on health outcomes, including metabolic indicators (e.g., TC, TG, LDL-C, HDL-C, FBG, HOMA-IR, BW,WC, BMI, SBP, DBP), inflammatory markers (e.g., IL-6 and TNF-α), fatigue and exercise endurance, seasonal upper respiratory infections, colds, sexual function, female menopausal symptoms, unstable angina as well as NSCLC and related complications. Adverse events included gastrointestinal symptoms and potential bleeding, but no serious adverse events were reported. However, there are several limitations in methodological quality across studies. Therefore, researchers should pay attention to the design and implementation of RCTs. Moreover, researchers need to comply with basic guidelines for reporting clinical trials so as to provide better evidence to support the healthcare needs of patients.

## Data Availability

The raw data supporting the conclusion of this article will be made available by the authors, without undue reservation.

## Abbreviations

ALT, alanine aminotransferase; AST, aspartate aminotransferase; ALP, alkaline phosphatase; ALB, albumin; BIL, bilirubin; BW, body weight; BMI, body mass index; BF%, body fat percentage; DBP, diastolic blood pressure; FPG, fasting plasma glucose; GGT, gamma-glutamyl transferase; HDL-C, high-density lipoprotein cholesterol; HbA1c, hemoglobin A1c; HOMA-IR, Homeostatic Model Assessment of Insulin Resistance scores; hs-CRP, high-sensitivity C-reactive protein; IL-6, interleukin 6; LDL-C, low-density lipoprotein cholesterol; MD, mean difference; Mets, metabolic syndrome; NSCLC, non-small cell lung cancer; OR, odds ratio; Qol, quality of life; RR, risk ratio; SBP, systolic blood pressure; SMD, standardized mean difference; SAURIs, seasonal acute upper respiratory infections; TC, total cholesterol; TG, triglycerides; T2DM, type 2 diabetes mellitus; TNF-α, tumor necrosis factor alpha; OGTT, oral glucose tolerance test results; WC, waist circumference.

## References

[B1] An X. Zhang A. L. Yang A. W. Lin L. Wu D. Guo X. (2011). Oral ginseng formulae for stable chronic obstructive pulmonary disease: A systematic review. Respir. Med. 105 (2), 165–176. 10.1016/j.rmed.2010.11.007 21146973

[B2] Antonelli M. Donelli D. Firenzuoli F. (2020). Ginseng integrative supplementation for seasonal acute upper respiratory infections: A systematic review and meta-analysis. Complementary Ther. Med. 52, 102457. 10.1016/j.ctim.2020.102457 PMC7305750 32951718

[B3] Aromataris E. Fernandez R. Godfrey C. M. Holly C. Khalil H. Tungpunkom P. (2015). Summarizing systematic reviews: Methodological development, conduct and reporting of an umbrella review approach. Int. J. Evidence-based Healthc. 13 (3), 132–140. 10.1097/XEB.0000000000000055 26360830

[B4] Bach H. V. Kim J. Myung S. K. Cho Y. A. (2016). Efficacy of ginseng supplements on fatigue and physical performance: A meta-analysis. J. Korean Med. Sci. 31 (12), 1879–1886. 10.3346/jkms.2016.31.12.1879 27822924 PMC5102849

[B5] Bahrke M. S. Morgan W. P. Stegner A. (2009). Is ginseng an ergogenic aid? Int. J. Sport Nutr. Exerc. Metabolism 19 (3), 298–322. 10.1123/ijsnem.19.3.298 19574616

[B6] Bak M. J. Jun M. Jeong W. S. (2012). Antioxidant and hepatoprotective effects of the red ginseng essential oil in H(2)O(2)-treated hepG2 cells and CCl(4)-treated mice. Int. J. Mol. Sci. 13 (2), 2314–2330. 10.3390/ijms13022314 22408456 PMC3292025

[B7] Castela Â. Costa C. (2016). Molecular mechanisms associated with diabetic endothelial-erectile dysfunction. Nat. Rev. Urol. 13 (5), 266–274. 10.1038/nrurol.2016.23 26878803

[B8] Chen G. Li H. Zhao Y. Zhu H. Cai E. Gao Y. (2017). Saponins from stems and leaves of Panax ginseng prevent obesity via regulating thermogenesis, lipogenesis and lipolysis in high-fat diet-induced obese C57BL/6 mice. Food Chem. Toxicol. Int. J. Publ. Br. Industrial Biol. Res. Assoc. 106, 393–403. 10.1016/j.fct.2017.06.012 28599882

[B9] Chen W. Yao P. Vong C. T. Li X. Chen Z. Xiao J. (2021). Ginseng: A bibliometric analysis of 40-year journey of global clinical trials. J. Adv. Res. 34, 187–197. 10.1016/j.jare.2020.07.016 35024190 PMC8655123

[B10] Chen X. (1996). Cardiovascular protection by ginsenosides and their nitric oxide releasing action. Clin. Exp. Pharmacol. Physiology 23 (8), 728–732. 10.1111/j.1440-1681.1996.tb01767.x 8886498

[B11] Chien P. F. W. Khan K. S. Siassakos D. (2012). Registration of systematic reviews: Prospero. BJOG Int. J. Obstetrics Gynaecol. 119 (8), 903–905. 10.1111/j.1471-0528.2011.03242.x 22703418

[B12] Coleman C. I. Hebert J. H. Reddy P. (2003). The effects of Panax ginseng on quality of life. J. Clin. Pharm. Ther. 28 (1), 5–15. 10.1046/j.1365-2710.2003.00467.x 12605613

[B13] Crichton M. Davidson A. R. Innerarity C. Marx W. Lohning A. Isenring E. (2022). Orally consumed ginger and human health: An umbrella review. Am. J. Clin. Nutr. 115 (6), 1511–1527. 10.1093/ajcn/nqac035 35147170 PMC9170469

[B14] Deng H. L. Zhang J. T. (1991). Anti-lipid peroxilative effect of ginsenoside Rb1 and Rg1. Chin. Med. J. 104 (5), 395–398. 1879209

[B15] Dong H. Ma J. Li T. Xiao Y. Zheng N. Liu J. (2017). Global deregulation of ginseng products may be a safety hazard to warfarin takers: Solid evidence of ginseng-warfarin interaction. Sci. Rep. 7 (1), 5813. 10.1038/s41598-017-05825-9 28725042 PMC5517508

[B16] Duan L. Xiong X. Hu J. Liu Y. Wang J. (2018). Efficacy and safety of oral panax notoginseng saponins for unstable angina patients: A meta-analysis and systematic review. Phytomedicine Int. J. Phytotherapy Phytopharm. 47, 23–33. 10.1016/j.phymed.2018.04.044 30166105

[B17] Duan Z. Deng J. Dong Y. Zhu C. Li W. Fan D. (2017). Anticancer effects of ginsenoside Rk3 on non-small cell lung cancer cells: *In vitro* and *in vivo* . Food & Funct. 8 (10), 3723–3736. 10.1039/c7fo00385d 28949353

[B18] Fong C. Alesi S. Mousa A. Moran L. J. Deed G. Grant S. (2022). Efficacy and safety of nutrient supplements for glycaemic control and insulin resistance in type 2 diabetes: An umbrella review and hierarchical evidence synthesis. Nutrients 14 (11), 2295. 10.3390/nu14112295 35684094 PMC9182772

[B19] Furuya-Kanamori L. Xu C. Lin L. Doan T. Chu H. Thalib L. (2020). P value-driven methods were underpowered to detect publication bias: Analysis of Cochrane review meta-analyses. J. Clin. Epidemiol. 118, 86–92. 10.1016/j.jclinepi.2019.11.011 31743750

[B20] Ghavami A. Ziaei R. Foshati S. Hojati Kermani M. A. Zare M. Amani R. (2020). Benefits and harms of ginseng supplementation on liver function? A systematic review and meta-analysis. Complementary Ther. Clin. Pract. 39, 101173. 10.1016/j.ctcp.2020.101173 32379697

[B21] Ghorbani Z. Mirghafourvand M. (2019). A meta-analysis of the efficacy of panax ginseng on menopausal women’s sexual function. Int. J. Women's Health Reproduction Sci. 7 (1), 124–133. 10.15296/ijwhr.2019.20

[B22] Guyatt G. Oxman A. D. Akl E. A. Kunz R. Vist G. Brozek J. (2011). GRADE guidelines: 1. Introduction-GRADE evidence profiles and summary of findings tables. J. Clin. Epidemiol. 64 (4), 383–394. 10.1016/j.jclinepi.2010.04.026 21195583

[B23] Hayashino Y. Noguchi Y. Fukui T. (2005). Systematic evaluation and comparison of statistical tests for publication bias. J. Epidemiol. 15 (6), 235–243. 10.2188/jea.15.235 16276033 PMC7904376

[B24] He Y. Yang J. Lv Y. Chen J. Yin F. Huang J. (2018). A review of ginseng clinical trials registered in the WHO international clinical trials registry platform. BioMed Res. Int. 2018, 1843142. 10.1155/2018/1843142 29546050 PMC5818925

[B25] Herrmann D. Sinnett P. Holmes J. Khan S. Koller C. Vassar M. (2017). Statistical controversies in clinical research: Publication bias evaluations are not routinely conducted in clinical oncology systematic reviews. Ann. Oncol. Official J. Eur. Soc. Med. Oncol. 28 (5), 931–937. 10.1093/annonc/mdw691 28039176

[B26] Huang J. Liu D. Wang Y. Liu L. Li J. Yuan J. (2022). Ginseng polysaccharides alter the gut microbiota and kynurenine/tryptophan ratio, potentiating the antitumour effect of antiprogrammed cell death 1/programmed cell death ligand 1 (anti-PD-1/PD-L1) immunotherapy. Gut 71 (4), 734–745. 10.1136/gutjnl-2020-321031 34006584 PMC8921579

[B27] Ikeuchi S. Minamida M. Nakamura T. Konishi M. Kamioka H. (2022). Exploratory systematic review and meta-analysis of *panax* genus plant ingestion evaluation in exercise endurance. Nutrients 14 (6), 1185. 10.3390/nu14061185 35334841 PMC8950061

[B28] Izzo A. A. Ernst E. (2009). Interactions between herbal medicines and prescribed drugs: An updated systematic review. Drugs 69 (13), 1777–1798. 10.2165/11317010-000000000-00000 19719333

[B29] Jang D. J. Lee M. S. Shin B. C. Lee Y. C. Ernst E. (2008). Red ginseng for treating erectile dysfunction: A systematic review. Br. J. Clin. Pharmacol. 66 (4), 444–450. 10.1111/j.1365-2125.2008.03236.x 18754850 PMC2561113

[B30] Kang S. Park S. J. Lee A. Y. Huang J. Chung H. Y. Im D. S. (2018). Ginsenoside Rg_3_ promotes inflammation resolution through M2 macrophage polarization. J. Ginseng Res. 42 (1), 68–74. 10.1016/j.jgr.2016.12.012 29348724 PMC5766702

[B31] Kim D. H. (2012). Chemical diversity of panax ginseng, panax quinquifolium, and panax notoginseng. J. Ginseng Res. 36 (1), 1–15. 10.5142/jgr.2012.36.1.1 23717099 PMC3659563

[B32] Lee H. W. Ang L. Lee M. S. (2022). Using ginseng for menopausal women's health care: A systematic review of randomized placebo-controlled trials. Complementary Ther. Clin. Pract. 48, 101615. 10.1016/j.ctcp.2022.101615 35691259

[B33] Lee H. W. Lee M. S. Kim T. H. Alraek T. Zaslawski C. Kim J. W. (2021). Ginseng for erectile dysfunction. Cochrane Database Syst. Rev. 4, 12654. 10.1002/14651858.CD012654.pub2 PMC8094213 33871063

[B34] Lee J. H. Min D. S. Lee C. W. Song K. H. Kim Y. S. Kim H. P. (2018). Ginsenosides from Korean red ginseng ameliorate lung inflammatory responses: Inhibition of the MAPKs/NF-κB/c-Fos pathways. J. Ginseng Res. 42 (4), 476–484. 10.1016/j.jgr.2017.05.005 30337808 PMC6187099

[B35] Leichsenring F. Steinert C. Rabung S. Ioannidis J. P. A. (2022). The efficacy of psychotherapies and pharmacotherapies for mental disorders in adults: An umbrella review and meta-analytic evaluation of recent meta-analyses. World Psychiatry Official J. World Psychiatric Assoc. (WPA) 21 (1), 133–145. 10.1002/wps.20941 PMC8751557 35015359

[B36] Leung K. W. Wong A. S. T. (2010). Pharmacology of ginsenosides: A literature review. Chin. Med. 5, 20. 10.1186/1749-8546-5-20 20537195 PMC2893180

[B37] Lev-Ari S. Starr A. N. Vexler A. Kalich-Philosoph L. Yoo H. S. Kwon K. R. (2021). Rh2-enriched Korean ginseng (Ginseng Rh2+) inhibits tumor growth and development of metastasis of non-small cell lung cancer. Food & Funct. 12 (17), 8068–8077. 10.1039/d1fo00643f 34286798

[B38] Li H. Huang N. Zhu W. Wu J. Yang X. Teng W. (2018). Modulation the crosstalk between tumor-associated macrophages and non-small cell lung cancer to inhibit tumor migration and invasion by ginsenoside Rh2. BMC Cancer 18 (1), 579. 10.1186/s12885-018-4299-4 29783929 PMC5963019

[B39] Li X. Liu J. Zuo T. T. Hu Y. Li Z. Wang H. D. (2022). Advances and challenges in ginseng research from 2011 to 2020: The phytochemistry, quality control, metabolism, and biosynthesis. Nat. Product. Rep. 39 (4), 875–909. 10.1039/d1np00071c 35128553

[B40] Liu Z. Qu C. Y. Li J. X. Wang Y. F. Li W. Wang C. Z. (2021). Hypoglycemic and hypolipidemic effects of malonyl ginsenosides from American ginseng (*Panax quinquefolius* L) on type 2 diabetic mice. ACS Omega 6 (49), 33652–33664. 10.1021/acsomega.1c04656 34926913 PMC8675029

[B41] Lü J. M. Yao Q. Chen C. (2009). Ginseng compounds: An update on their molecular mechanisms and medical applications. Curr. Vasc. Pharmacol. 7 (3), 293–302. 10.2174/157016109788340767 19601854 PMC2928028

[B42] Lundh A. Lexchin J. Mintzes B. Schroll J. B. Bero L. (2018). Industry sponsorship and research outcome: Systematic review with meta-analysis. Intensive Care Med. 44 (10), 1603–1612. 10.1007/s00134-018-5293-7 30132025

[B43] Luo W. T. Huang T. W. (2022). Effects of ginseng on cancer-related fatigue: A systematic review and meta-analysis of randomized controlled trials. Cancer Nurs. 46, 120–127. 10.1097/NCC.0000000000001068 35184068

[B44] Miraghajani M. Hadi A. Hajishafiee M. Arab A. Ghaedi E. Moody V. (2020). The effects of ginseng supplementation on anthropometric indices and body composition: A systematic review and meta-analysis. J. Herb. Med. 23, 100379. 10.1016/j.hermed.2020.100379

[B45] Mohammadi H. Hadi A. Kord-Varkaneh H. Arab A. Afshari M. Ferguson A. J. R. (2019). Effects of ginseng supplementation on selected markers of inflammation: A systematic review and meta-analysis. Phytotherapy Res. PTR 33 (8), 1991–2001. 10.1002/ptr.6399 31161680

[B46] Moher D. Liberati A. Tetzlaff J. Altman D. G. PRISMA Group (2009). Preferred reporting items for systematic reviews and meta-analyses: The PRISMA statement. BMJ Clin. Res. ed.) 339, b2535. 10.1136/bmj.b2535 PMC2714657 19622551

[B47] Murugesan M. Mathiyalagan R. Boopathi V. Kong B. M. Choi S. K. Lee C. S. (2022). Production of minor ginsenoside CK from major ginsenosides by biotransformation and its advances in targeted delivery to tumor tissues using nanoformulations. Nanomater. (Basel, Switz. 12 (19), 3427. 10.3390/nano12193427 PMC9565578 36234555

[B48] Naseri K. Saadati S. Sadeghi A. Asbaghi O. Ghaemi F. Zafarani F. (2022). The efficacy of ginseng (panax) on human prediabetes and type 2 diabetes mellitus: A systematic review and meta-analysis. Nutrients 14 (12), 2401. 10.3390/nu14122401 35745129 PMC9227417

[B49] Page M. J. Shamseer L. Tricco A. C. (2018). Registration of systematic reviews in PROSPERO: 30,000 records and counting. Syst. Rev. 7 (1), 32. 10.1186/s13643-018-0699-4 29463298 PMC5819709

[B50] Park S. H. Chung S. Chung M. Y. Choi H. K. Hwang J. T. Park J. H. (2022). Effects of *Panax ginseng* on hyperglycemia, hypertension, and hyperlipidemia: A systematic review and meta-analysis. J. Ginseng Res. 46 (2), 188–205. 10.1016/j.jgr.2021.10.002 35509826 PMC9058846

[B51] Park S. K. Hyun S. H. Park C. K. Kwak Y. S. Jang Y. J. (2021). The antioxidant activities of Korean red ginseng (*Panax ginseng*) and ginsenosides: A systemic review through *in vivo* and clinical trials. J. Ginseng Res. 45 (1), 41–47. 10.1016/j.jgr.2020.09.006 33437155 PMC7790892

[B52] Qin N. Gong Q. H. Wei L. W. Wu Q. Huang X. N. (2008). Total ginsenosides inhibit the right ventricular hypertrophy induced by monocrotaline in rats. Biol. Pharm. Bull. 31 (8), 1530–1535. 10.1248/bpb.31.1530 18670084

[B53] Qiu X. Wang C. (2016). Literature searches in the conduct of systematic reviews and evaluations. Shanghai Archives Psychiatry 28 (3), 154–159. 10.11919/j.issn.1002-0829.215008 PMC5434301 28638185

[B54] Qu L. Zou W. Wang Y. Wang M. (2018). European regulation model for herbal medicine: The assessment of the EU monograph and the safety and efficacy evaluation in marketing authorization or registration in Member States. Phytomedicine Int. J. Phytotherapy Phytopharm. 42, 219–225. 10.1016/j.phymed.2018.03.048 29655689

[B55] Rabasco A. McKay D. Smits J. A. Powers M. B. Meuret A. E. McGrath P. B. (2022). Psychosocial treatment for panic disorder: An umbrella review of systematic reviews and meta-analyses. J. Anxiety Disord. 86, 102528. 10.1016/j.janxdis.2022.102528 35063924

[B56] Rombey T. Puljak L. Allers K. Ruano J. Pieper D. (2020). Inconsistent views among systematic review authors toward publishing protocols as peer-reviewed articles: An international survey. J. Clin. Epidemiol. 123, 9–17. 10.1016/j.jclinepi.2020.03.010 32201257

[B57] Saboori S. Falahi E. Yousefi Rad E. Asbaghi O. Khosroshahi M. Z. (2019). Effects of ginseng on C-reactive protein level: A systematic review and meta-analysis of clinical trials. Complementary Ther. Med. 45, 98–103. 10.1016/j.ctim.2019.05.021 31331589

[B58] Schulz K. F. Altman D. G. Moher D. CONSORT Group (2010). CONSORT 2010 statement: Updated guidelines for reporting parallel group randomized trials. Ann. Intern. Med. 152 (11), 726–732. 10.7326/0003-4819-152-11-201006010-00232 20335313

[B59] Seida J. K. Durec T. Kuhle S. (2011). North American (panax quinquefolius) and asian ginseng (panax ginseng) preparations for prevention of the common cold in healthy adults: A systematic review. Evidence-based Complementary Altern. Med. ECAM 2011, 282151. 10.1093/ecam/nep068 PMC3136130 19592479

[B60] Sha'ari N. Woon L. S. C. Sidi H. Das S. Bousman C. A. Mohamed Saini S. (2021). Beneficial effects of natural products on female sexual dysfunction: A systematic review and meta-analysis. Phytomedicine Int. J. Phytotherapy Phytopharm. 93, 153760. 10.1016/j.phymed.2021.153760 34638031

[B61] Shea B. J. Reeves B. C. Wells G. Thuku M. Hamel C. Moran J. (2017). Amstar 2: A critical appraisal tool for systematic reviews that include randomised or non-randomised studies of healthcare interventions, or both. BMJ Clin. Res. ed.) 358, j4008. 10.1136/bmj.j4008 PMC5833365 28935701

[B62] Shergis J. L. Zhang A. L. Zhou W. Xue C. C. (2013). Panax ginseng in randomised controlled trials: A systematic review. Phytotherapy Res. PTR 27 (7), 949–965. 10.1002/ptr.4832 22969004

[B63] Sideri S. Papageorgiou S. N. Eliades T. (2018). Registration in the international prospective register of systematic reviews (PROSPERO) of systematic review protocols was associated with increased review quality. J. Clin. Epidemiol. 100, 103–110. 10.1016/j.jclinepi.2018.01.003 29339215

[B64] Song X. Chen J. Sakwiwatkul K. Li R. Hu S. (2010). Enhancement of immune responses to influenza vaccine (H3N2) by ginsenoside Re. Int. Immunopharmacol. 10 (3), 351–356. 10.1016/j.intimp.2009.12.009 20034596

[B65] Sun Y. Liu Y. Chen K. (2016). Roles and mechanisms of ginsenoside in cardiovascular diseases: Progress and perspectives. Sci. China Life Sci. 59 (3), 292–298. 10.1007/s11427-016-5007-8 26798041

[B66] Tan S. J. Li N. Zhou F. Dong Q. T. Zhang X. D. Chen B. C. (2014). Ginsenoside Rb1 improves energy metabolism in the skeletal muscle of an animal model of postoperative fatigue syndrome. J. Surg. Res. 191 (2), 344–349. 10.1016/j.jss.2014.04.042 24881470

[B67] Tan S. Zhou F. Li N. Dong Q. Zhang X. Ye X. (2013). Anti-fatigue effect of ginsenoside Rb1 on postoperative fatigue syndrome induced by major small intestinal resection in rat. Biol. Pharm. Bull. 36 (10), 1634–1639. 10.1248/bpb.b13-00522 23924778

[B68] Wang D. S. Wang J. M. Zhang F. R. Lei F. J. Wen X. Song J. (2022). Ameliorative effects of malonyl ginsenoside from *Panax ginseng* on glucose-lipid metabolism and insulin resistance via IRS1/PI3K/akt and AMPK signaling pathways in type 2 diabetic mice. Am. J. Chin. Med. 50 (3), 863–882. 10.1142/S0192415X22500367 35282802

[B69] Wang L. Huang Y. Yin G. Wang J. Wang P. Chen Z. Y. (2020). Antimicrobial activities of Asian ginseng, American ginseng, and notoginseng. Phytotherapy Res. PTR 34 (6), 1226–1236. 10.1002/ptr.6605 31885119

[B70] Xie J. T. Shao Z. H. Vanden Hoek T. L. Chang W. T. Li J. Mehendale S. (2006). Antioxidant effects of ginsenoside Re in cardiomyocytes. Eur. J. Pharmacol. 532 (3), 201–207. 10.1016/j.ejphar.2006.01.001 16497296

[B71] Yang Q. Y. Lai X. D. Ouyang J. Yang J. D. (2018). Effects of Ginsenoside Rg3 on fatigue resistance and SIRT1 in aged rats. Toxicology 409, 144–151. 10.1016/j.tox.2018.08.010 30144466

[B72] Yang Z. Dan W. Li Y. Zhou X. Liu T. Shi C. (2022). Untargeted metabolomics analysis of the anti-diabetic effect of Red ginseng extract in Type 2 diabetes Mellitus rats based on UHPLC-MS/MS. Biomed. Pharmacother. = Biomedecine Pharmacother. 146, 112495. 10.1016/j.biopha.2021.112495 34891123

[B73] Yin J. Zhang H. Ye J. (2008). Traditional Chinese medicine in treatment of metabolic syndrome. Endocr. Metabolic Immune Disord. Drug Targets 8 (2), 99–111. 10.2174/187153008784534330 PMC2467395 18537696

[B74] Ying A. Yu Q. T. Guo L. Zhang W. S. Liu J. F. Li Y. (2018). Structural-activity relationship of ginsenosides from steamed ginseng in the treatment of erectile dysfunction. Am. J. Chin. Med. 46 (1), 137–155. 10.1142/S0192415X18500088 29298510

[B75] Yoon S. J. Kim S. K. Lee N. Y. Choi Y. R. Kim H. S. Gupta H. (2021). Effect of Korean red ginseng on metabolic syndrome. J. Ginseng Res. 45 (3), 380–389. 10.1016/j.jgr.2020.11.002 34025131 PMC8134847

[B76] Yu T. Rhee M. H. Lee J. Kim S. H. Yang Y. Kim H. G. (2016). Ginsenoside rc from Korean red ginseng (panax ginseng C.A. Meyer) attenuates inflammatory symptoms of gastritis, hepatitis and arthritis. Am. J. Chin. Med. 44 (3), 595–615. 10.1142/S0192415X16500336 27109153

[B77] Yun T. K. (2001). Brief introduction of Panax ginseng C.A. Meyer. J. Korean Med. Sci. 16, S3–S5. 10.3346/jkms.2001.16.S.S3 11748372 PMC3202213

[B78] Zhang X. Deng J. Tang Y. Guan X. Chen X. Fan J. (2022). Zingiberaceae plants/curcumin consumption and multiple health outcomes: An umbrella review of systematic reviews and meta-analyses of randomized controlled trials in humans. Phytotherapy Res. PTR 36, 3080–3101. 10.1002/ptr.7500 35623903

[B79] Zhou P. Lu S. Luo Y. Wang S. Yang K. Zhai Y. (2017). Attenuation of TNF-α-Induced inflammatory injury in endothelial cells by ginsenoside Rb1 via inhibiting NF-κB, JNK and p38 signaling pathways. Front. Pharmacol. 8, 464. 10.3389/fphar.2017.00464 28824425 PMC5540891

[B80] Zhu H. Liu H. Zhu J. H. Wang S. Y. Zhou S. S. Kong M. (2021). Efficacy of ginseng and its ingredients as adjuvants to chemotherapy in non-small cell lung cancer. Food & Funct. 12 (5), 2225–2241. 10.1039/d0fo03341c 33595586

[B81] Zhu J. Xu X. Zhang X. Zhuo Y. Chen S. Zhong C. (2022). Efficacy of ginseng supplements on disease-related fatigue: A systematic review and meta-analysis. Medicine 101 (26), 29767. 10.1097/MD.0000000000029767 PMC9239648 35776997

