# Supplementary material for: Ginseng and health outcomes: an umbrella review
Source: Front Pharmacol. 2023 Jul 3;14:1069268. doi: 10.3389/fphar.2023.1069268 (PMC10351045; doi:10.3389/fphar.2023.1069268)
Supplement: Supplementary file 1 [file Table1.DOCX]

Supplementary Material

# Supplementary Table S1. Electronic Database Search Strategies

| **Pubmed**  197results  (((meta analys*[Title/Abstract]) OR (systematic review*[Title/Abstract])) OR (("Meta-Analysis" [Publication Type]) OR "Meta-Analysis as Topic"[Mesh])) AND (((ginseng[Title/Abstract]) OR (Panax[Title/Abstract])) OR ("Panax"[Mesh]))AND ("0001/01/01"[PDAT]:"2022/07/31"[PDAT]) |
| --- |
| **Embase**  341results  ((('panax'/exp) OR (ginseng:ab,ti,kw OR panax:ab,ti,kw)) AND (('meta analys*':ti,ab,kw OR 'systematic review*':ti,ab,kw) or ('meta analysis'/exp))) AND AND [<1966-2022]/py |
| **Scopus**  588 results  ((TITLE-ABS-KEY ("meta analys*")  OR TITLE-ABS-KEY ("systematic review*"))) AND ((TITLE-ABS-KEY(panax) OR TITLE-ABS-KEY(panax))) |
| **Cochrane**  5 results  ((MeSH descriptor: [panax] explode all trees) OR (panax):ti,ab,kw OR (panax):ti,ab,kw (Word variations have been searched)) in Cochrane Reviews |
| **CNKI**  63 results  SU=('西洋参' +'人参'+'三七') AND SU=('荟萃分析' +'系统评价'+'Meta分析') |
| **Wang fang database**  39 results  (((题名或关键词=人参) OR 题名或关键词=西洋参) OR 题名或关键词=三七) AND (((题名或关键词=meta 分析) OR 题名或关键词=系统评价) OR 题名或关键词=荟萃分析) |
