# Supplementary material for: Ginseng and health outcomes: an umbrella review
Source: Front Pharmacol. 2023 Jul 3;14:1069268. doi: 10.3389/fphar.2023.1069268 (PMC10351045; doi:10.3389/fphar.2023.1069268)
Supplement: Supplementary file 2 [file Table2.docx]

Supplementary Material

# Supplementary Table S2 GRADE profile of ginseng supplementation for health outcomes

| **Outcomes** | **Population** | **Intervention/ comparation** | **Number of RCTs** | **Sample size（I/C）** | **Effect metrics** | **Estimates** | **95%CI** | **I^2^** | **P value** | **Risk of bias** | **Inconsistency** | **Indirectness** | **Imprecision** | **Publication bias** | **GRADE level** |
| --- | --- | --- | --- | --- | --- | --- | --- | --- | --- | --- | --- | --- | --- | --- | --- |
| **Blood lipids** | | | | | | | | | | | | | | | |
| TC (Park et al., 2022) | Metabolic diseases | Ginseng /placebo | 18 | 803 (398/405) | MD | -0.17 | -0.28, -0.05 | 0% | 0.005 | Serious ^a^ | Not serious | Not serious | Not serious | Not serious | ⨁⨁⨁◯  Moderate |
| TC (Naseri et al., 2022) | Prediabetes and T2DM | Ginseng /placebo | 13 | NR | WMD | -5.77 | -11.53, -0.01 | 80.8% | 0.04 | Serious ^a^ | Very serious ^g^ | Not serious | Not serious | Not serious | ⨁◯◯◯  Very low |
| TC (Duan et al., 2018) | Unstable angina patients | PNS+CM/CM | 4 | 312 (156/156) | MD | -0.79 | -0.93, -0.66 | 96% | <0.00001 | Serious ^a^ | Very serious ^g^ | Not serious | Not serious | Not mention | ⨁◯◯◯  Very low |
| TG (Park et al., 2022) | Metabolic diseases | Ginseng /placebo | 17 | 741  (366/375) | MD | -0.11 | -0.21, -0.01 | 0% | 0.03 | Serious ^a^ | Not serious | Not serious | Not serious | Not serious | ⨁⨁⨁◯  Moderate |
| TG (Naseri et al., 2022) | Prediabetes and T2DM | Ginseng /placebo | 12 | NR | WMD | 6.05 | -4.37, 16.48 | 73.5% | 0.225 | Serious ^a^ | Serious ^b^ | Not serious | Serious ^d^ | Not serious | ⨁◯◯◯  Very low |
| TG (Duan et al., 2018) | Unstable angina patients | PNS+CM/CM | 4 | 312  (156/156) | MD | -0.23 | -0.29, -0.17 | 98% | <0.00001 | Serious ^a^ | Very serious ^g^ | Not serious | Not serious | Not mention | ⨁◯◯◯  Very low |
| LDL-C (Park et al., 2022) | Metabolic diseases | Ginseng /placebo | 16 | 693  (343/350) | MD | -0.24 | -0.36, -0.13 | 0% | <0.0001 | Serious ^a^ | Not serious | Not serious | Not serious | Not serious | ⨁⨁⨁◯  Moderate |
| LDL-C (Naseri et al., 2022) | Prediabetes and T2DM | Ginseng /placebo | 13 | NR | WMD | -4.16 | -8.98, 0.65 | 78.2% | 0.09 | Serious ^a^ | Very serious ^g^ | Not serious | Serious ^d^ | Not serious | ⨁◯◯◯  Very low |
| LDL-C (Duan et al., 2018) | Unstable angina patients | PNS+CM/CM | 4 | 312  (156/156) | MD | -0.77 | -0.91, -0.63 | 92% | <0.00001 | Serious ^a^ | Very serious ^g^ | Not serious | Not serious | Not mention | ⨁◯◯◯  Very low |
| HDL-C (Naseri et al., 2022) | Prediabetes and T2DM | Ginseng /placebo | 13 | NR | WMD | -1.28 | -5.58, 3.01 | 96.9% | 0.557 | Serious ^a^ | Very serious ^g^ | Not serious | Serious ^d^ | Not serious | ⨁◯◯◯  Very low |
| HDL ([Lian Duan](https://pubmed.ncbi.nlm.nih.gov/?sort=pubdate&size=50&term=Duan+L&cauthor_id=30166105)，2018(Duan et al., 2018)) | Unstable angina patients | PNS+CM/CM | 3 | 200(NR) | MD | 0.30 | 0.24, 0.36 | 93% | <0.00001 | Serious ^a^ | Very serious ^g^ | Not serious | Serious ^e^ | Not mention | ⨁◯◯◯  Very low |
| **Blood glucose and insulin secretion** | | | | | | | | | | | | | | | |
| FPG (Naseri et al., 2022) | Prediabetes and T2DM | Ginseng /placebo | 13 | 1077  (567/510) | WMD | -7.03 | -10.89, -3.17 | 91% | ＜0.001 | Serious ^a^ | Very serious ^g^ | Not serious | Not serious | Serious ^c^ | ⨁◯◯◯  Very low |
| OGTT (Naseri et al., 2022) | Prediabetes and T2DM | Ginseng /placebo | 8 | 347  (190/157) | WMD | -6.81 | -16.77, 3.14 | 66.3% | 0.18 | Serious ^a^ | Serious ^b^ | Not serious | Serious ^d^ | Not serious | ⨁◯◯◯  Very low |
| HbA1c (Naseri et al., 2022) | Prediabetes and T2DM | Ginseng /placebo | 12 | 914  (478/436) | WMD | -0.04 | -0.16, 0.07 | 82.9% | 0.449 | Serious ^a^ | Serious ^b^ | Not serious | Serious ^d^ | Not serious | ⨁⨁◯◯  Low |
| HOMA-IR (Naseri et al., 2022) | Prediabetes and T2DM | Ginseng /placebo | 10 | 390  (211/179) | WMD | -0.44 | -0.84, -0.04 | 67.6% | 0.001 | Serious ^a^ | Serious ^b^ | Not serious | Not serious | Not serious | ⨁⨁◯◯  Low |
| Fasting insulin levels (Naseri et al., 2022) | Prediabetes and T2DM | Ginseng /placebo | 16 | 570  (306/264) | WMD | -0.13 | -1.18, 0.90 | 89.8% | 0.0000 | Serious ^a^ | Very serious ^g^ | Not serious | Serious ^d^ | Not serious | ⨁◯◯◯  Very low |
| **Anthropometric indices and body composition** | | | | | | | | | | | | | | | |
| BF% body fat (Park et al., 2022) | Metabolic diseases | Ginseng /placebo | 4 | 106  (53/53) | MD | -2.11 | -3.98, -0.23 | 0% | 0.03 | Serious ^a^ | Not serious | Not serious | Serious ^e^ | Not serious | ⨁⨁◯◯  Low |
| BF% (Miraghajani et al., 2020) | Adults | Ginseng /placebo | 4 | 137  (68/69) | MD | -1.137% | -4.006, 1.732 | 97.7% | 0.437 | Serious ^a^ | Very serious ^g^ | Not serious | Very serious ^d, e^ | Not serious | ⨁◯◯◯  Very low |
| BW (Naseri et al., 2022) | Prediabetes and T2DM | Ginseng /placebo | 5 | 480  (244/236) | WMD | -0.54 | -2.54, 1.46 | 0.0% | 0.598 | Serious ^a^ | Not serious | Not serious | Serious ^d^ | Not serious | ⨁⨁◯◯  Low |
| BW (Miraghajani et al., 2020) | Adults | Ginseng /placebo | 5 | 159  (78/79) | MD | -0.038 | -0.665, 0.589 | 0% | 0.905 | Serious ^a^ | Not serious | Not serious | Very serious ^d, e^ | Not serious | ⨁◯◯◯  Very low |
| BMI (Naseri et al., 2022) | Prediabetes and T2DM | Ginseng /placebo | 9 | 648  (344/304) | WMD | 0.05 | -0.26, 0.38 | 0.0% | 0.717 | Serious ^a^ | Not serious | Not serious | Serious ^d^ | Not serious | ⨁⨁◯◯  Low |
| BMI (Miraghajani et al., 2020) | Adults | Ginseng /placebo | 9 | 378  (190/188) | MD | 0.103 | -0.080, 0.286 | 39.4% | 0.268 | Serious ^a^ | Not serious | Not serious | Serious ^d^ | Not serious | ⨁⨁◯◯ Low |
| WC (Naseri et al., 2022) | Prediabetes and T2DM | Ginseng /placebo | 3 | 72  (54/18) | WMD | 0.05 | -1.16, 1.27 | 0.0% | 0.929 | Serious ^a^ | Not serious | Not serious | Serious ^d^ | Not serious | ⨁⨁◯◯  Low |
| **Blood pressure and heart rate** | | | | | | | | | | | | | | | |
| SBP (Park et al., 2022) | Metabolic diseases | ginseng /placebo | 15 | 702  (347/355) | MD | -3.23 | -4.19, -2.27 | 33% | <0.00001 | Serious ^a^ | Not serious | Not serious | Not serious | Serious ^c^ | ⨁⨁◯◯  Low |
| SBP (Naseri et al., 2022) | Prediabetes and T2MD | Ginseng /placebo | 10 | 420  (227/193) | WMD | -2.78 | -6.97, 1.40 | 87.4% | 0.000 | Serious ^a^ | Very serious ^g^ | Not serious | Serious ^d^ | Not serious | ⨁◯◯◯  Very low |
| DBP (Park et al., 2022) | Metabolic diseases | Ginseng /placebo | 14 | 657  (326/331) | MD | -1.48 | -3.18, 0.21 | 52% | 0.09 | Serious ^a^ | Serious ^b^ | Not serious | Serious ^d^ | Serious ^c^ | ⨁◯◯◯  Very low |
| DBP (Naseri et al., 2022) | Prediabetes and T2DM | Ginseng /placebo | 10 | 420  (227/193) | WMD | -0.24 | -1.88, 1.39 | 63.4% | 0.003 | Serious ^a^ | Serious ^b^ | Not serious | Serious ^d^ | Not serious | ⨁◯◯◯  Very low |
| Heart Rate (Naseri et al., 2022) | Prediabetes and T2DM | Ginseng /placebo | 3 | 224  (116/128) | WMD | 2.65 | 2.20, 3.09 | 1.2% | 0.363 | Serious ^a^ | Not serious | Not serious | Serious ^e^ | Not serious | ⨁⨁◯◯  Low |
| **Inflammatory markers and adipocytokines** | | | | | | | | | | | | | | | |
| hs‐CRP (Mohammadi et al., 2019) | Adults | Ginseng /placebo | 6 | 308  (154/154) | MD | -0.125 | -0.597, 0.347 | 97.4% | 0.604 | Serious ^a^ | Very serious ^g^ | Serious ^f^ | Serious ^d^ | Serious ^c^ | ⨁◯◯◯  Very low |
| IL‐6 (Mohammadi et al., 2019) | Adults | Ginseng /placebo | 4 | 227  (115/112) | MD | -0.265 | -0.396, -0.135 | 97.7% | ＜0.001 | Serious ^a^ | Very serious ^g^ | Not serious | Serious ^e^ | Not serious | ⨁◯◯◯  Very low |
| IL-6 levels (Naseri et al., 2022) | Prediabetes and T2DM | Ginseng /placebo | 6 | 232  (132/100) | WMD | -1.22 | -1.68, -0.75 | 27.3% | 0.23 | Serious ^a^ | Not serious | Not serious | Serious ^e^ | Serious ^c^ | ⨁◯◯◯  Very low |
| TNF‐α (Mohammadi et al., 2019) | Adults | Ginseng /placebo | 4 | 156  (80/76） | MD | -2.471 | -2.904,-2.039 | 72.4% | <0.001 | Serious ^a^ | Serious ^b^ | Not serious | Serious ^e^ | Not serious | ⨁◯◯◯  Very low |
| TNF-α (Naseri et al., 2022) | Prediabetes and T2DM | Ginseng /placebo | 6 | 232  (132/100) | WMD | 2.15 | 0.66, 3.63 | 0% | 0.92 | Serious ^a^ | Not serious | Not serious | Very serious ^d, e^ | Serious ^c^ | ⨁◯◯◯  Very low |
| CRP (Saboori et al., 2019) | Adults with any healthy status | Ginseng /placebo | 9 | 420(NR) | WMD | -0.1 | -0.26, 0.1 | 81.95% | 0.27 | Serious ^a^ | Very serious ^g^ | Not serious | Serious ^d^ | Not serious | ⨁◯◯◯  Very low |
| CRP (Naseri et al., 2022) | Prediabetes and T2DM | Ginseng /placebo | 6 | 232  (132/100) | WMD | -0.10 | -0.61, 0.41 | 55.8% | 0.046 | Serious ^a^ | Serious ^b^ | Not serious | Very serious ^d, e^ | Not serious | ⨁◯◯◯  Very low |
| Adiponectin (Naseri et al., 2022) | Prediabetes and T2DM | Ginseng /placebo | 3 | 72  (54/18) | WMD | -0.27 | -1.41, 0.86 | 0% | 0.906 | Serious ^a^ | Not serious | Not serious | Very serious ^d, e^ | Not serious | ⨁◯◯◯  Very low |
| Leptin (Naseri et al., 2022) | Prediabetes and T2DM | Ginseng /placebo | 3 | 72  (54/18) | WMD | -0.67 | -2.01, 0.65 | 0% | 0.847 | Serious ^a^ | Not serious | Not serious | Very serious ^d, e^ | Not serious | ⨁◯◯◯  Very low |
| **Fatigue and physical function** | | | | | | | | | | | | | | | |
| Fatigue reduction (Bach et al., 2016) | Patients or healthy people | Ginseng /placebo | 4 | 458  (NR) | SMD | 0.34 | 0.16, 0.52 | 5.2% | NR | Not serious | Not serious | Serious ^f^ | Not serious | Not mention | ⨁⨁⨁◯  Moderate |
| physical performance enhancement (Bach et al., 2016) | Patients or healthy people | Ginseng /placebo | 8 | 212  (126/86) | SMD | -0.01 | -0.29, 0.27 | 0% | NR | Serious ^a^ | Not serious | Serious ^f^ | Very serious ^d, e^ | Not mention | ⨁◯◯◯  Very low |
| Exercise Endurance (Ikeuchi et al., 2022) | Adults with no healthy problems | Panax genus plants or ginsenoside /placebo | 5 | 123  (59/64） | SMD | 0.58 | 0.22, 0.95 | 0% | 0.002 | Serious ^a^ | Not serious | Not serious | Serious ^e^ | Not mention | ⨁⨁◯◯  Low |
| Disease-related fatigue (Zhu et al., 2022) | Participants with underlying diseases | Ginseng /placebo | 12 | 1298  (NR) | SMD | -0.33 | -0.44, -0.22 | 35% | <0.00001 | Serious ^a^ | Not serious | Serious ^f^ | Not serious | Not serious | ⨁⨁⨁◯  Moderate |
| Cancer-Related Fatigue (Luo and Huang, 2022) | Patients with cancer | Ginseng /placebo | 4 | 530  (NR) | SMD | -0.21 | -0.42, 0.00 | 17% | 0.05 | Serious ^a^ | Not serious | Serious ^f^ | Not serious | Not mention | ⨁⨁◯◯  Low |
| **Sexual function and menopausal symptoms** | | | | | | | | | | | | | | | |
| Menopausal symptoms (Lee et al., 2022) | Menopausal women | Ginseng /placebo | 3 | 515  (258/257) | SMD | -0.40 | -0.73, -0.07 | 55% | 0.02 | Serious ^a^ | Serious ^b^ | Not serious | Not serious | Not mention | ⨁⨁◯◯  Low |
| Hot flashes (Lee et al., 2022) | Menopausal women | Ginseng /placebo | 3 | 515  (258/257) | SMD | -0.34 | -0.66, -0.01 | 53% | 0.04 | Serious ^a^ | Serious ^b^ | Not serious | Not serious | Not mention | ⨁⨁◯◯  Low |
| Sexual function (Lee et al., 2022) | Menopausal women | Ginseng /placebo | 3 | 491  (246/245) | SMD | 0.31 | -0.30, 0.92 | 84% | 0.32 | Serious ^a^ | Very serious ^g^ | Not serious | Serious ^d^ | Not mention | ⨁◯◯◯  Very low |
| QoL (Lee et al., 2022) | Menopausal women | Ginseng /placebo | 3 | 515  (258/257) | SMD | -0.31 | -0.61, -0.01 | 47% | 0.05 | Serious ^a^ | Not serious | Not serious | Not serious | Not mention | ⨁⨁⨁◯  Moderate |
| IIEF-EF (Lee et al., 2021) | Adult men with ED | Ginseng /placebo | 3 | 245  (145/100) | MD | 3.52 | 1.79, 5.25 | 0% | <0.0001 | Serious ^a^ | Not serious | Not serious | Very serious ^d, e^ | Not mention | ⨁◯◯◯  Very low |
| IIEF-5 (Lee et al., 2021) | Adult men with ED | Ginseng /placebo | 3 | 236  (140/96) | MD | 2.39 | 0.89, 3.88 | 0% | 0.002 | Serious ^a^ | Not serious | Not serious | Very serious ^d, e^ | Not mention | ⨁◯◯◯  Very low |
| Ability to have intercourse reported by participants (Lee et al., 2021) | Adult men with ED | Ginseng /placebo | 6 | 349  (185/164) | RR | 2.55 | 1.76, 3.69 | 23% | <0.00001 | Serious ^a^ | Not serious | Serious ^f^ | Not serious | Not mention | ⨁⨁◯◯  Low |
| Sexual satisfaction (Lee et al., 2021) | Adult men with ED | Ginseng /placebo | 3 | 245  (145/100) | MD | 1.19 | 0.41, 1.97 | 0% | 0.003 | Serious ^a^ | Not serious | Not serious | Very serious ^d, e^ | Not mention | ⨁◯◯◯  Very low |
| Response rate of ED (Jang et al., 2008) | ED patients | Ginseng /placebo | 6 | 349  (185/164) | RR | 2.40 | 1.65, 3.51 | 22% | <0.00001 | Serious ^a^ | Not serious | Serious ^f^ | Not serious | Not mention | ⨁⨁◯◯  Low |
| Sexual functions (Jang et al., 2008) | ED patients | Red ginseng /placebo | 3 | 151  (76/75) | SMD | 0.79 | 0.46, 1.12 | 0% | <0.00001 | Serious ^a^ | Not serious | Serious ^f^ | Serious ^e^ | Not mention | ⨁◯◯◯  Very low |
| Sexual functions (Sha'ari et al., 2021) | Female sexual dysfunction patients | Ginseng /placebo | 3 | 156  (78/78) | SMD | 0.279 | -0.315, 0.874 | 71.46% | 0.355 | Serious ^a^ | Serious ^b^ | Serious ^f^ | Very serious ^d, e^ | Not mention | ⨁◯◯◯  Very low |
| Sexual Arousal (Sha'ari et al., 2021) | Female sexual dysfunction patients | Ginseng /placebo | 3 | 156  (78/78) | SMD | 0.536 | 0.108, 0.965 | 44.18% | 0.014 | Serious ^a^ | Not serious | Serious ^f^ | Serious ^e^ | Not mention | ⨁◯◯◯  Very low |
| Sexual Desire (Sha'ari et al., 2021) | Female sexual dysfunction patients | Ginseng /placebo | 3 | 156  (78/78) | SMD | 0.585 | 0.266, 0.904 | 0% | <0.001 | Serious ^a^ | Not serious | Serious ^f^ | Serious ^e^ | Not mention | ⨁◯◯◯  Very low |
| Sexual functions (Ghorbani and Mirghafourvand, 2019) | Menopausal women | Ginseng /placebo | 6 | 531  (289/287) | SMD | 0.26 | -0.24, 0.76 | 81% | 0.30 | Serious ^a^ | Very serious ^g^ | Serious ^f^ | Not serious | Not mention | ⨁◯◯◯  Very low |
| **Respiratory system diseases** | | | | | | | | | | | | | | | |
| Incidence of SAURIs (Antonelli et al., 2020) | Adults | Ginseng extract/placebo | 9 | 1550  (NR) | RR | 0.69 | 0.52, 0.90 | 58.4% | NR | Serious ^a^ | Serious ^b^ | Not serious | Not serious | Serious ^c^ | ⨁◯◯◯  Very low |
| duration of disease symptoms (Antonelli et al., 2020) | Adults with SAURIs | Ginseng extract/placebo | 7 | 1152  (563/589) | MD | -2.58 | -5.40, 0.24 | 64.0% | NR | Serious ^a^ | Serious ^b^ | Not serious | Serious ^d^ | Not mention | ⨁⨁◯◯  Low |
| Incidence of common colds and other ARIs (Seida et al., 2011) | Adults | Ginseng /placebo | 5 | 747  (363/384) | RR | 0.70 | 0.48, 1.02 | 68.5% | 0.06 | Serious ^a^ | Serious | Not serious | Serious ^d^ | Not mention | ⨁◯◯◯  Very low |
| Duration of disease symptoms (Seida et al., 2011) | Adults with colds | Ginseng /placebo | 2 | 189  (80/109) | MD | -6.18 | -9.00, -3.36 | 0% | ＜0.0001 | Not serious | Not serious | Not serious | Serious ^e^ | Not mention | ⨁⨁⨁◯  Moderate |
| ORR (Zhu et al., 2021) | NSCLC | Ginseng and its ingredients+chemotherapy/chemotherapy | 22 | 1713  (871/842) | RR | 1.35 | 1.21, 1.50 | 0% | <0.00001 | Serious ^a^ | Not serious | Not serious | Not serious | Not serious | ⨁⨁⨁◯  Moderate |
| DCR (Zhu et al., 2021) | NSCLC | Ginseng and its ingredients+chemotherapy/chemotherapy | 22 | 1713  (871/842) | RR | 1.20 | 1.14, 1.28 | 21% | <0.00001 | Serious ^a^ | Not serious | Not serious | Not serious | Serious ^c^ | ⨁⨁◯◯  Low |
| QoL(Zhu et al., 2021) | NSCLC | Ginseng and its ingredients+CT/CT | 14 | 1082  (550/532) | RR | 1.31 | 1.22, 1.41 | 0% | <0.00001 | Serious ^a^ | Not serious | Not serious | Not serious | Serious ^c^ | ⨁⨁◯◯  Low |
| Leucopenia (Zhu et al., 2021) | NSCLC | Ginseng and its ingredients+CT/CT | 12 | 1199  (597/602) | RR | 0.59 | 0.50, 0.70 | 17% | <0.00001 | Serious ^a^ | Not serious | Not serious | Not serious | Not serious | ⨁⨁⨁◯  Moderate |
| Thrombocytopenia (Zhu et al., 2021) | NSCLC | Ginseng and its ingredients+CT/CT | 9 | 989  (491/498) | RR | 0.53 | 0.37, 0.76 | 0% | 0.0005 | Serious ^a^ | Not serious | Not serious | Not serious | Not serious | ⨁⨁⨁◯  Moderate |
| Hemoglobin decline (Zhu et al., 2021) | NSCLC | Ginseng and its ingredients+CT/CT | 5 | 664  (328/336) | RR | 0.63 | 0.27, 1.45 | 0% | 0.27 | Serious ^a^ | Not serious | Not serious | Serious ^d^ | Not mention | ⨁⨁◯◯  Low |
| Myelosuppression (Zhu et al., 2021) | NSCLC | Ginseng and its ingredients+CT/CT | 4 | 380  (190/190) | RR | 0.30 | 0.17, 0.53 | 0% | <0.00001 | Serious ^a^ | Not serious | Not serious | Not serious | Not mention | ⨁⨁⨁◯  Moderate |
| Hepatotoxicity (Zhu et al., 2021) | NSCLC | Ginseng and its ingredients+CT/CT | 11 | 835  (425/410) | RR | 0.67 | 0.53, 0.87 | 0% | 0.02 | Serious ^a^ | Not serious | Not serious | Not serious | Not serious | ⨁⨁⨁◯  Moderate |
| Alopecia (Zhu et al., 2021) | NSCLC | Ginseng and its ingredients+CT/CT | 4 | 321  (162/159) | RR | 0.73 | 0.52, 1.02 | 0% | 0.07 | Serious ^a^ | Not serious | Not serious | Serious ^d^ | Not mention | ⨁⨁◯◯  Low |
| Diarrhea (Zhu et al., 2021) | NSCLC | Ginseng and its ingredients+CT/CT | 4 | 339  (171/168) | RR | 0.42 | 0.19,0.96 | 0% | 0.04 | Serious ^a^ | Not serious | Not serious | Not serious | Not mention | ⨁⨁⨁◯  Moderate |
| Nausea and vomiting (Zhu et al., 2021) | NSCLC | Ginseng and its ingredients+CT/CT | 12 | 901  (455/446) | RR | 0.67 | 0.53, 0.86 | 0% | 0.001 | Serious ^a^ | Not serious | Not serious | Not serious | Not serious | ⨁⨁⨁◯  Moderate |
| one-year survival rate (Zhu et al., 2021) | NSCLC | Ginseng and its ingredients+CT/CT | 7 | 843  (417/426) | RR | 1.35 | 1.13, 1.60 | 0% | 0.0008 | Serious ^a^ | Not serious | Not serious | Not serious | Not mention | ⨁⨁⨁◯  Moderate |
| Two-year survival rate (Zhu et al., 2021) | NSCLC | Ginseng and its ingredients+CT/CT | 4 | 219  (113/106) | RR | 3.21 | 1.51, 6.81 | 45% | 0.02 | Serious ^a^ | Not serious ^b^ | Not serious | Serious ^e^ | Not mention | ⨁◯◯◯  Very low |
| CD3+ (Zhu et al., 2021) | NSCLC | Ginseng and its ingredients+CT/CT | 7 | 660  (336/324) | SMD | 1.76 | 1.21, 2.31 | 89% | <0.00001 | Serious ^a^ | Serious ^b^ | Not serious | Not serious | Not mention | ⨁⨁◯◯  Low |
| CD4+ (Zhu et al., 2021) | NSCLC | Ginseng and its ingredients+CT/CT | 6 | 354  (177/177) | SMD | 2.02 | 1.26, 2.77 | 77.45% | <0.00001 | Serious ^a^ | Very serious ^g^ | Not serious | Not serious | Not mention | ⨁◯◯◯  Very low |
| CD8+ (Zhu et al., 2021) | NSCLC | Ginseng and its ingredients+CT/CT | 5 | 510  (261/249) | SMD | 1.12 | -0.52, 2.76 | 98% | 0.18 | Serious ^a^ | Very serious ^g^ | Not serious | Not serious | Not mention | ⨁◯◯◯  Very low |
| CD4+/CD8+(Zhu et al., 2021) | NSCLC | Ginseng and its ingredients+CT/CT | 8 | 672  (343/329) | SMD | 1.39 | 0.63, 2.16 | 95% | 0.0004 | Serious ^a^ | Very serious ^g^ | Not serious | Not serious | Not serious | ⨁◯◯◯  Very low |
| **Liver function** | | | | | | | | | | | | | | | |
| ALT (Ghavami et al., 2020) | Adults | Ginseng /placebo | 14 | 992  (494/498) | WMD | 0.04 | -1.80, 1.89 | 49.3% | 0.964 | Serious ^a^ | Not serious | Not serious | Serious ^d^ | Not serious | ⨁◯◯◯  Very low |
| AST (Ghavami et al., 2020) | Adults | Ginseng /placebo | 12 | 876  (436/440) | WMD | 0.16 | -1.19, 1.52 | 53.6% | 0.811 | Serious ^a^ | Serious ^b^ | Not serious | Serious ^d^ | Not serious | ⨁◯◯◯  Very low |
| ALP (Ghavami et al., 2020) | Adults | Ginseng /placebo | 8 | 382  (192/190) | WMD | -0.03 | -0.23, 0.17 | 0% | 0.766 | Serious ^a^ | Not serious | Not serious | Serious ^d^ | Not serious | ⨁⨁◯◯  Low |
| GGT (Ghavami et al., 2020) | Adults | Ginseng /placebo | 12 | 609  (305/304) | WMD | 1.04 | -0.69, 2.7 | 0% | 0.237 | Serious ^a^ | Not serious | Not serious | Serious ^d^ | Not serious | ⨁⨁◯◯  Low |
| ALB (Ghavami et al., 2020) | Adults | Ginseng /placebo | 8 | 430  (216/214) | WMD | -0.23 | -0.52, 0.07 | 94.7% | 0.138 | Serious ^a^ | Very serious ^g^ | Not serious | Serious ^d^ | Not serious | ⨁◯◯◯  Very low |
| BIL (Ghavami et al., 2020) | Adults | Ginseng /placebo | 8 | NR | WMD | 0.07 | 0.02, 0.12 | 0% | 0.004 | Serious ^a^ | Not serious | Not serious | Not serious | Not serious | ⨁⨁⨁◯  Moderate |
| ALT( (Naseri et al., 2022) | Prediabetes and T2DM | Ginseng /placebo | 7 | 269  (153/116) | WMD | 0.62 | -1.90, 3.15 | 46% | 0.085 | Serious ^a^ | Not serious | Not serious | Very serious ^d, e^ | Not serious | ⨁◯◯◯  Very low |
| AST (Naseri et al., 2022) | Prediabetes and T2DM | Ginseng /placebo | 5 | 165  (98/67) | WMD | −0.28 | −1.77, 1.19 | 22.3% | 0.272 | Serious ^a^ | Not serious | Not serious | Very serious ^d, e^ | Not serious | ⨁◯◯◯  Very low |
| GGT (Naseri et al., 2022) | Prediabetes and T2DM | Ginseng /placebo | 3 | 72  (54/18) | WMD | 2.03 | −6.22, 10.28 | 20.3% | 0.285 | Serious ^a^ | Not serious | Not serious | Very serious ^d, e^ | Not serious | ⨁◯◯◯  Very low |
| **Cardiovascular disease** | | | | | | | | | | | | | | | |
| End point(Duan et al., 2018) | Unstable angina patients | PNS+CM/CM | 3 | 1360  (680/680) | OR | 0.38 | 0.22, 0.63 | 0% | 0.0002 | Serious ^a^ | Not serious | Serious ^f^ | Not serious | Not mention | ⨁⨁◯◯  Low |
| Frequency of angina attack (Duan et al., 2018) | Unstable angina patients | PNS+CM/CM | 7 | 766  (398/368) | MD | -2.07 | -2.20, -1.94 | 97% | <0.00001 | Serious ^a^ | Very serious ^g^ | Not serious | Not serious | Not mention | ⨁◯◯◯  Very low |
| Duration of angina attack (Duan et al., 2018) | Unstable angina patients | PNS+CM/CM | 4 | 436  (218/218) | MD | -1.88 | -2.03, -1.72 | 0% | <0.00001 | Serious ^a^ | Not serious | Not serious | Not serious | Not mention | ⨁⨁⨁◯  Moderate |
| Dosage of nitroglyceriN (Duan et al., 2018) | Unstable angina patients | PNS+CM/CM | 2 | 212  (106/106) | MD | -1.13 | -1.70, -0.56 | 0% | <0.0001 | Serious ^a^ | Not serious | Not serious | Serious ^e^ | Not mention | ⨁⨁◯◯  Low |
| ECG (Duan et al., 2018) | Unstable angina patients | PNS+CM/CM | 8 | 698  (349/349) | OR | 3.09 | 0.97, 6.95 | 32% | <0.00001 | Serious ^a^ | Serious ^b^ | Not serious | Not serious | Not mention | ⨁⨁◯◯  Low |

Abbreviations: MD: mean difference；SMD standard mean difference；RR: relative risk; OR: odd risk; CT：chemotherapy; CM: conventional therapy; NASLC: Non-small cell lung cancer; T2DM: type 2 diabetes mellitus; ED:  erectile dysfunction; BF%: body fat percentage; BW: body weight; WC: waist circumference; BMI: Body Mass Index; FPG: fasting plasma glucose; OGTT: oral glucose tolerance test; HbA1c: hemoglobin A1c; HOMA-IR: homeostatic model assessment of insulin resistance; TG: triglyceride; TC: total cholesterol; LDL-C: low-density lipoprotein cholesterol; HDL-C: high-density lipoprotein cholesterol; SBP: systolic blood pressure; DBP: diastolic blood pressure; HR: heart rate; CRP: C-reactive protein; hs-CRP: high-sensitive C-Reactive Protein; IL-6: interlukin-6; TNF-α: tumor necrosis factor-α; ALT: alanine aminotransferase; AST: aspartate aminotransferase; GGT: gamma-glutamyl transferase; Qol: qulity of life; ORR: overall response rate; DCR:  disease control rate; SAURIs: [seasonal acute upper respiratory infections](https://pubmed.ncbi.nlm.nih.gov/32951718/).

^a^ The included study had an unclear risk of selection, performance, detection, and reporting biases; ^b^ 50% ≤ I^2^ < 75%; ^c^ Funnel plot or Egger's or Begg's tests indicated asymmetry; ^d^ 95 % Cl includes invalid line; ^e^ Sample size <300; ^f^ Inconsistent measuring tools; ^g^ I^2^ ≥ 75%.

**Rererences**

Antonelli, M., Donelli, D., and Firenzuoli, F. (2020). Ginseng integrative supplementation for seasonal acute upper respiratory infections: A systematic review and meta-analysis. *Complementary Therapies In Medicine* 52**,** 102457. doi: 10.1016/j.ctim.2020.102457.

Bach, H.V., Kim, J., Myung, S.K., and Cho, Y.A. (2016). Efficacy of Ginseng Supplements on Fatigue and Physical Performance: a Meta-analysis. *Journal of Korean Medical Science* 31(12)**,** 1879-1886. doi: 10.3346/jkms.2016.31.12.1879.

Duan, L., Xiong, X., Hu, J., Liu, Y., and Wang, J. (2018). Efficacy and safety of oral Panax notoginseng saponins for unstable angina patients: A meta-analysis and systematic review. *Phytomedicine : International Journal of Phytotherapy and Phytopharmacology* 47**,** 23-33. doi: 10.1016/j.phymed.2018.04.044.

Ghavami, A., Ziaei, R., Foshati, S., Hojati Kermani, M.A., Zare, M., and Amani, R. (2020). Benefits and harms of ginseng supplementation on liver function? A systematic review and meta-analysis. *Complementary Therapies In Clinical Practice* 39**,** 101173. doi: 10.1016/j.ctcp.2020.101173.

Ghorbani, Z., and Mirghafourvand, M. (2019). A meta-analysis of the efficacy of panax ginseng on menopausal women’s sexual function. *International Journal of Women's Health and Reproduction Sciences* 7(1)**,** 124-133. doi: 10.15296/ijwhr.2019.20.

Ikeuchi, S., Minamida, M., Nakamura, T., Konishi, M., and Kamioka, H. (2022). Exploratory Systematic Review and Meta-Analysis of Genus Plant Ingestion Evaluation in Exercise Endurance. *Nutrients* 14(6)**,** 1185. doi: 10.3390/nu14061185.

Jang, D.-J., Lee, M.S., Shin, B.-C., Lee, Y.-C., and Ernst, E. (2008). Red ginseng for treating erectile dysfunction: a systematic review. *British Journal of Clinical Pharmacology* 66(4)**,** 444-450. doi: 10.1111/j.1365-2125.2008.03236.x.

Lee, H.W., Ang, L., and Lee, M.S. (2022). Using ginseng for menopausal women's health care: A systematic review of randomized placebo-controlled trials. *Complementary Therapies In Clinical Practice* 48**,** 101615. doi: 10.1016/j.ctcp.2022.101615.

Lee, H.W., Lee, M.S., Kim, T.-H., Alraek, T., Zaslawski, C., Kim, J.W., et al. (2021). Ginseng for erectile dysfunction. *The Cochrane Database of Systematic Reviews* 4**,** CD012654. doi: 10.1002/14651858.CD012654.pub2.

Luo, W.-T., and Huang, T.-W. (2022). Effects of Ginseng on Cancer-Related Fatigue: A Systematic Review and Meta-analysis of Randomized Controlled Trials. *Cancer Nursing*. doi: 10.1097/NCC.0000000000001068.

Miraghajani, M., Hadi, A., Hajishafiee, M., Arab, A., Ghaedi, E., and Moody, V. (2020). The effects of ginseng supplementation on anthropometric indices and body composition: A systematic review and meta-analysis. *Journal of Herbal Medicine* 23**,** 100379. doi: 10.1016/j.hermed.2020.100379.

Mohammadi, H., Hadi, A., Kord-Varkaneh, H., Arab, A., Afshari, M., Ferguson, A.J.R., et al. (2019). Effects of ginseng supplementation on selected markers of inflammation: A systematic review and meta-analysis. *Phytotherapy Research : PTR* 33(8)**,** 1991-2001. doi: 10.1002/ptr.6399.

Naseri, K., Saadati, S., Sadeghi, A., Asbaghi, O., Ghaemi, F., Zafarani, F., et al. (2022). The Efficacy of Ginseng (Panax) on Human Prediabetes and Type 2 Diabetes Mellitus: A Systematic Review and Meta-Analysis. *Nutrients* 14(12)**,** 2401. doi: 10.3390/nu14122401.

Park, S.H., Chung, S., Chung, M.-Y., Choi, H.-K., Hwang, J.-T., and Park, J.H. (2022). Effects of on hyperglycemia, hypertension, and hyperlipidemia: A systematic review and meta-analysis. *Journal of Ginseng Research* 46(2)**,** 188-205. doi: 10.1016/j.jgr.2021.10.002.

Saboori, S., Falahi, E., Yousefi Rad, E., Asbaghi, O., and Khosroshahi, M.Z. (2019). Effects of ginseng on C-reactive protein level: A systematic review and meta-analysis of clinical trials. *Complementary Therapies In Medicine* 45**,** 98-103. doi: 10.1016/j.ctim.2019.05.021.

Seida, J.K., Durec, T., and Kuhle, S. (2011). North American (Panax quinquefolius) and Asian Ginseng (Panax ginseng) Preparations for Prevention of the Common Cold in Healthy Adults: A Systematic Review. *Evidence-based Complementary and Alternative Medicine : ECAM* 2011**,** 282151. doi: 10.1093/ecam/nep068.

Sha'ari, N., Woon, L.S.-C., Sidi, H., Das, S., Bousman, C.A., and Mohamed Saini, S. (2021). Beneficial effects of natural products on female sexual dysfunction: A systematic review and meta-analysis. *Phytomedicine : International Journal of Phytotherapy and Phytopharmacology* 93**,** 153760. doi: 10.1016/j.phymed.2021.153760.

Zhu, H., Liu, H., Zhu, J.-H., Wang, S.-Y., Zhou, S.-S., Kong, M., et al. (2021). Efficacy of ginseng and its ingredients as adjuvants to chemotherapy in non-small cell lung cancer. *Food & Function* 12(5)**,** 2225-2241. doi: 10.1039/d0fo03341c.

Zhu, J., Xu, X., Zhang, X., Zhuo, Y., Chen, S., Zhong, C., et al. (2022). Efficacy of ginseng supplements on disease-related fatigue: A systematic review and meta-analysis. *Medicine* 101(26)**,** e29767. doi: 10.1097/MD.0000000000029767.
